# Supplementary material for: A CAG repeat-targeting artificial miRNA lowers the mutant huntingtin level in the YAC128 model of Huntington's disease
Source: Mol Ther Nucleic Acids. 2022 May 5;28:702–15. doi: 10.1016/j.omtn.2022.04.031 (PMC9126840; doi:10.1016/j.omtn.2022.04.031)
Supplement: Document S1. Figures S1–S6 and Tables S1–S4 [file mmc1.pdf]

OMTN, Volume *28*

Supplemental information

A CAG repeat-targeting artificial miRNA lowers the mutant huntingtin level in the YAC128 model of Huntington's disease

Anna Kotowska-Zimmer, Lukasz Przybyl, Marianna Pewinska, Joanna Suszynska-Zajczyk, Dorota Wronka, Maciej Figiel, and Marta Olejniczak

SUPPLEMENTAL INFORMATION

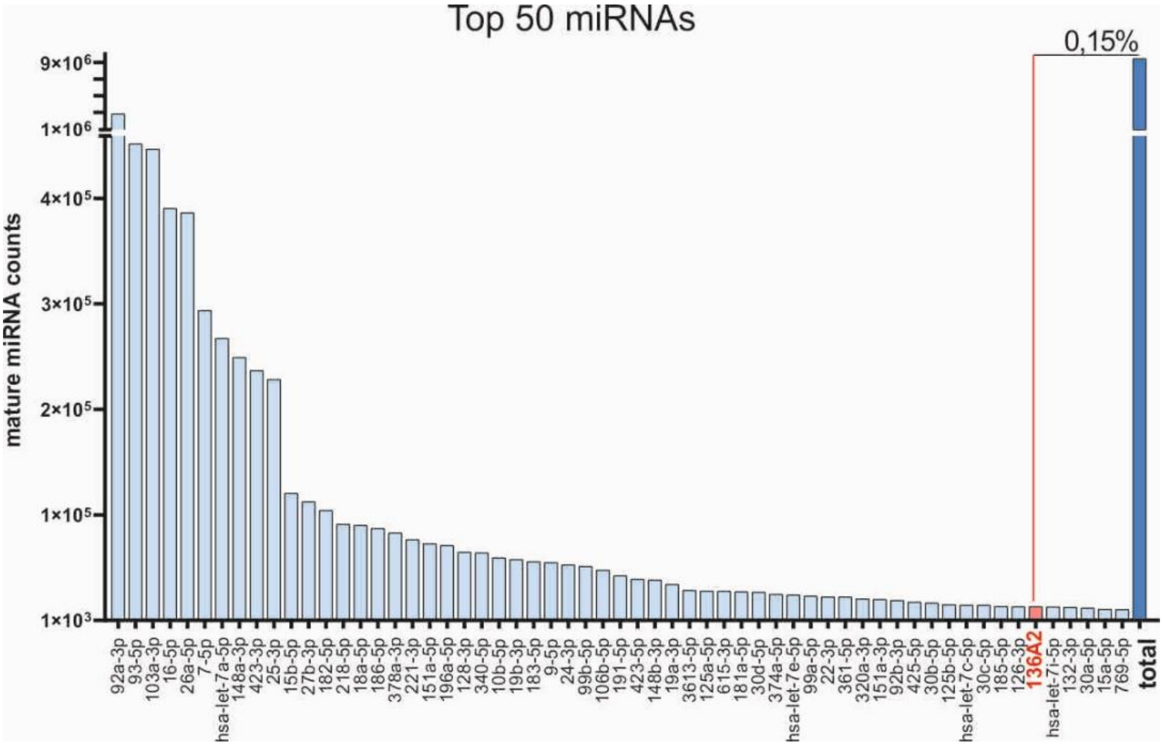

Supplemental Figure S1. Small RNA sequencing analysis of amiR136-A2-treated HEK293T cells. Top 50 most abundant miRNAs. The red bar indicates the mature amiR136-A2 count, and the last bar indicates the total mature miRNA count.

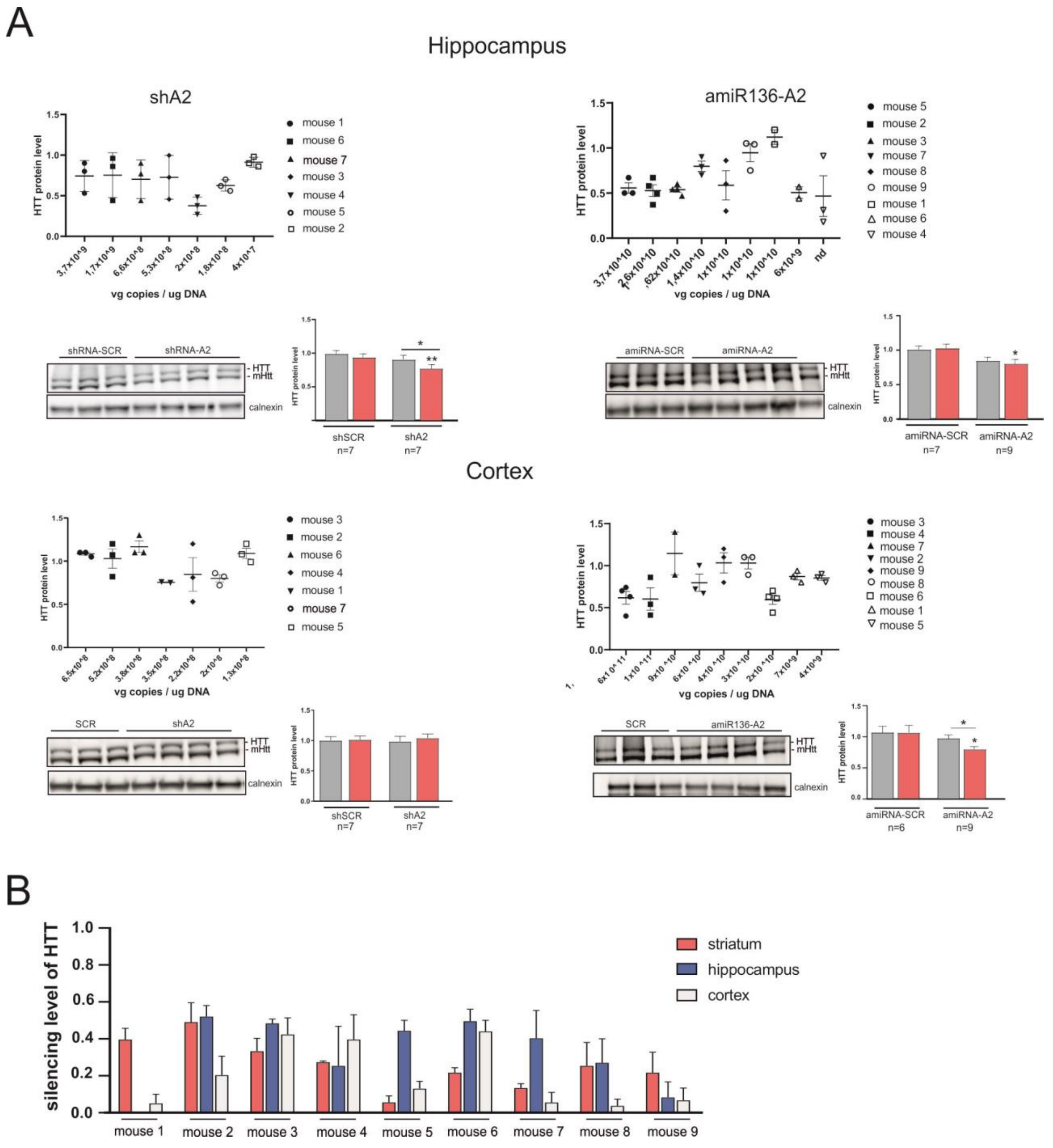

Supplemental Figure S2. Analysis of HTT protein silencing and AAV5 vector genome copies in the hippocampus and cortex of YAC128 mice. (A) qPCR to determine AAV5 genome copies (gc) in the brain structures of shA2- and amiR136-A2-injected mice (n=7 and n=9, respectively), one month post injection. Primers specific for the H1 and CAG promoters were used, and the gc values were calculated based on the standard curve. Western blot analysis of the HTT protein level. (B) Comparison of silencing efficiency in the striatum, hippocampus and cortex in amiR136-A2-treated mice. Signal intensities of the protein bands were normalized to those of calnexin and compared using Student's t-test. The bars on the graph indicate the mean protein levels ± SEMs. P values are indicated by asterisks (*p < 0.03, **p < 0.002).

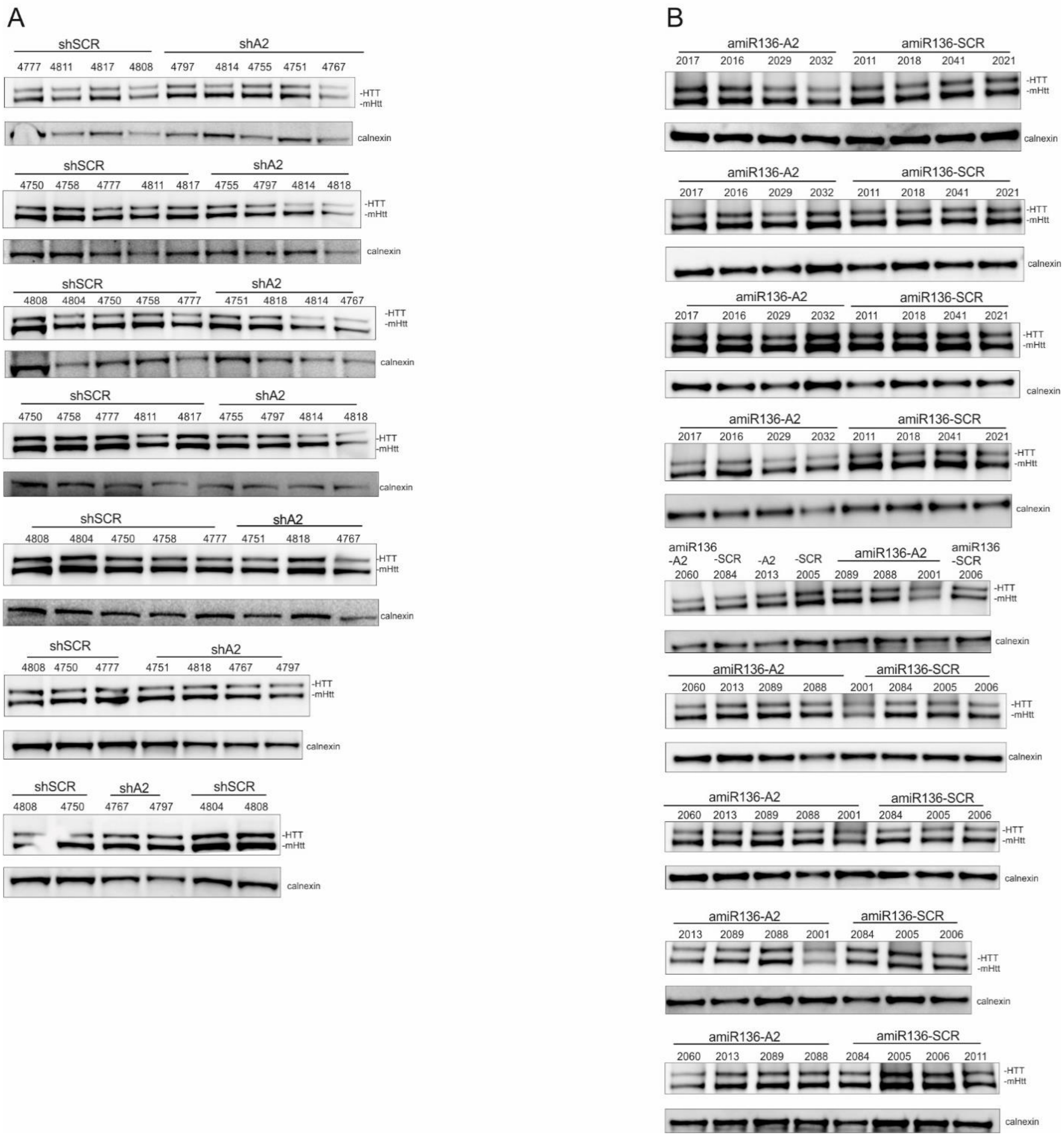

Supplemental Figure S3. Western blots used for quantification of HTT suppression in the striatum one month post injection of (A)AAV5-shA2 and (B) amiR136-A2.

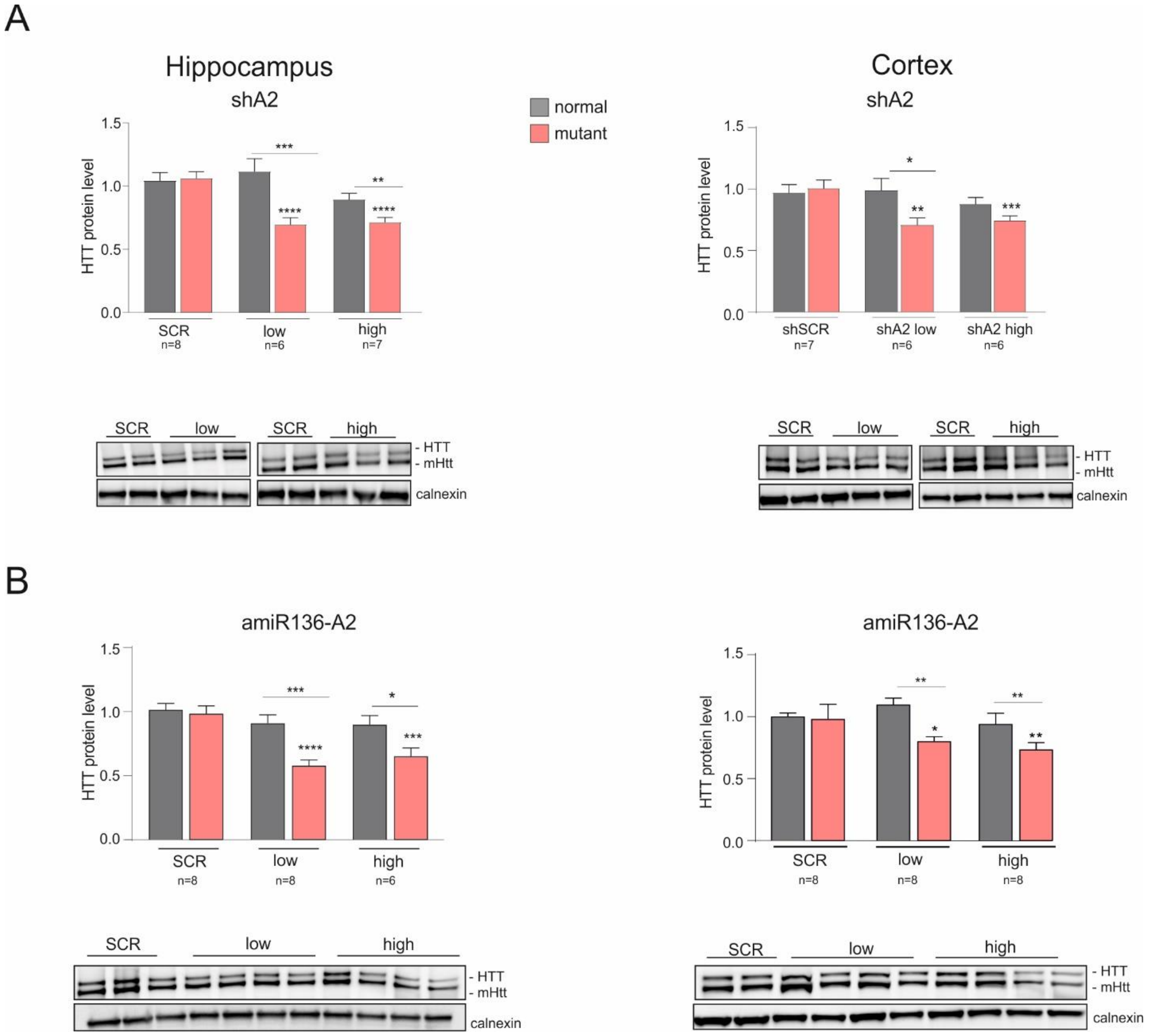

Supplemental Figure S4. Long-term analysis of the efficacy and allele selectivity of shA2 and amiR136-A2 in the hippocampus and cortex in treated mice. (**A**) Western blot analysis of the HTT protein level in the hippocampus and cortex 20 weeks post injection with AAV5-shA2. The shSCR construct was used as the reference control. (**B**) Western blot analysis of the HTT protein level in the hippocampus and cortex 20 weeks post injection with AAV5-amiR136-A2. The amiR136-SCR construct was used as the reference control. The bars on the graph indicate the mean protein levels ± SEMs. P values are indicated by asterisks (*p < 0.03, **p < 0.002, ***p < 0.0002, ****p<0.0001).

A

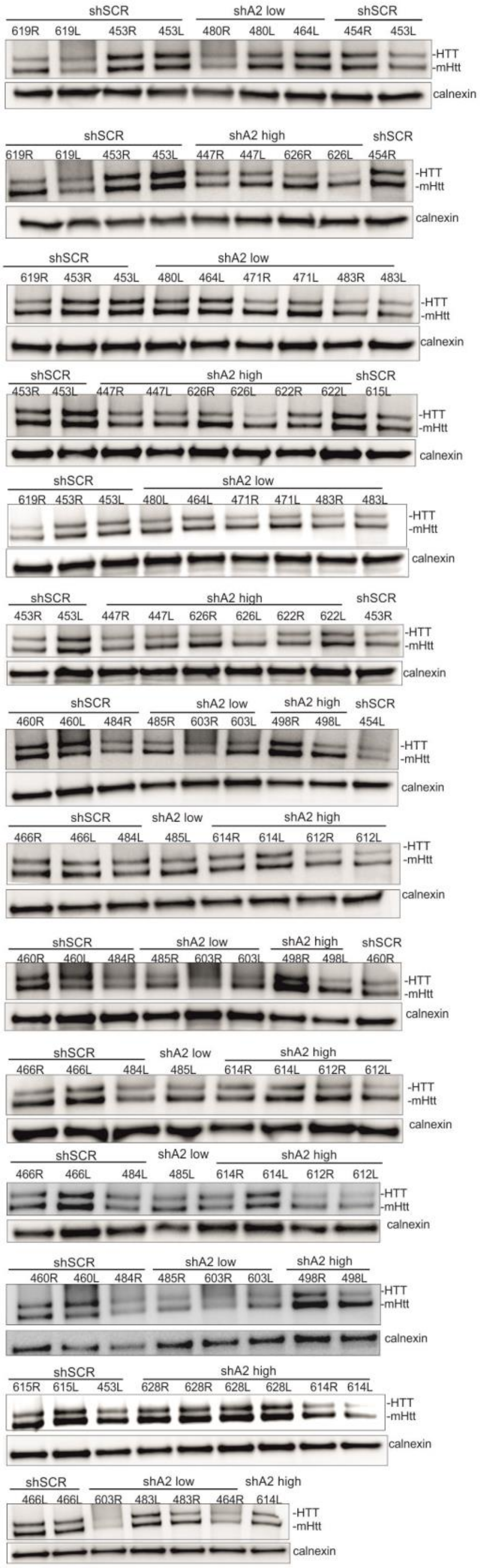

shSCR shA2 low shSCR
619R 619L 453R 453L 480R 480L 464L 454R 453L
-HTT
-mHtt
calnexin
shSCR shA2 high shSCR
619R 619L 453R 453L 447R 447L 626R 626L 454R
shSCR shA2 low
619R 453R 453L 480L 464L 471R 471L 483R 483L
shSCR shA2 high shSCR
453R 453L 447R 447L 626R 626L 622R 622L 615L
shSCR shA2 low
619R 453R 453L 480L 464L 471R 471L 483R 483L
shSCR shA2 high shSCR
453R 453L 447R 447L 626R 626L 622R 622L 453R
shSCR shA2 low shA2 high shSCR
460R 460L 484R 485R 603R 603L 498R 498L 454L
shSCR shA2 low shA2 high
466R 466L 484L 485L 614R 614L 612R 612L
shSCR shA2 low shA2 high shSCR
460R 460L 484R 485R 603R 603L 498R 498L 460R
shSCR shA2 low shA2 high
466R 466L 484L 485L 614R 614L 612R 612L
shSCR shA2 low shA2 high
466R 466L 484L 485L 614R 614L 612R 612L
shSCR shA2 low shA2 high
460R 460L 484R 485R 603R 603L 498R 498L
shSCR shA2 high
615R 615L 453L 628R 628R 628L 628L 614R 614L
shSCR shA2 low shA2 high
466L 466L 603R 483L 483R 464R 614L

B

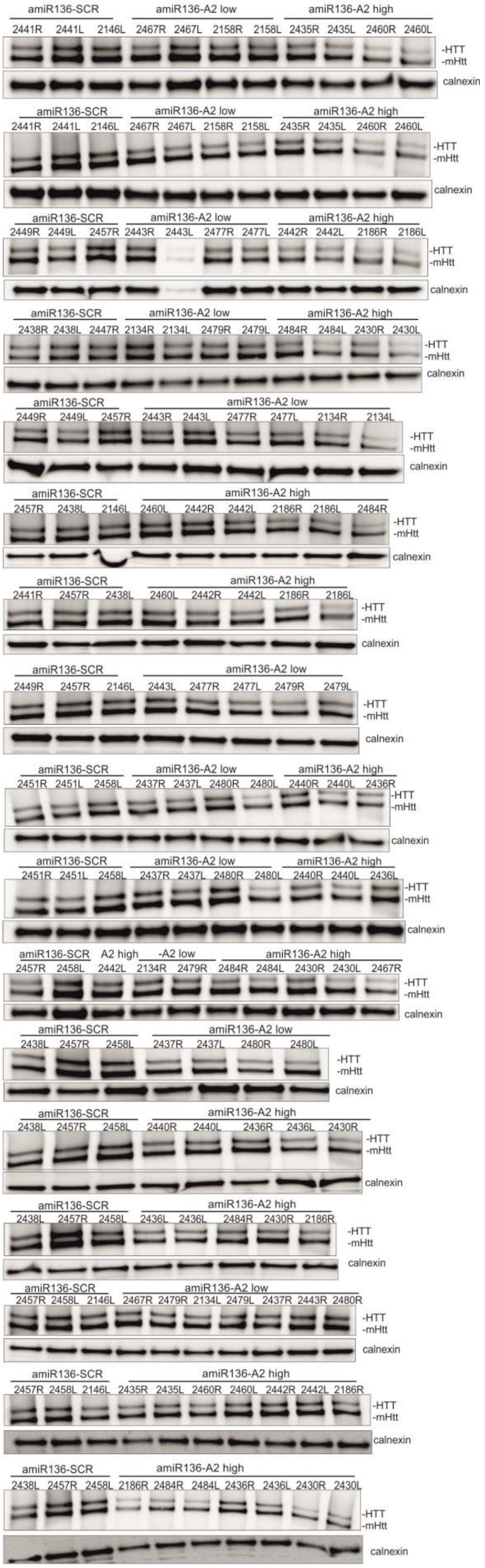

amiR136-SCR amiR136-A2 low amiR136-A2 high
2441R 2441L 2146L 2467R 2467L 2158R 2158L 2435R 2435L 2460R 2460L
-HTT
-mHtt
calnexin
amiR136-SCR amiR136-A2 low amiR136-A2 high
2441R 2441L 2146L 2467R 2467L 2158R 2158L 2435R 2435L 2460R 2460L
amiR136-SCR amiR136-A2 low amiR136-A2 high
2449R 2449L 2457R 2443R 2443L 2477R 2477L 2442R 2442L 2186R 2186L
amiR136-SCR amiR136-A2 low amiR136-A2 high
2438R 2438L 2447R 2134R 2134L 2479R 2479L 2484R 2484L 2430R 2430L
amiR136-SCR amiR136-A2 low
2449R 2449L 2457R 2443R 2443L 2477R 2477L 2134R 2134L
amiR136-SCR amiR136-A2 high
2457R 2438L 2146L 2460L 2442R 2442L 2186R 2186L 2484R
amiR136-SCR amiR136-A2 high
2441R 2457R 2438L 2460L 2442R 2442L 2186R 2186L
amiR136-SCR amiR136-A2 low
2449R 2457R 2146L 2443L 2477R 2477L 2479R 2479L
amiR136-SCR amiR136-A2 low amiR136-A2 high
2451R 2451L 2458L 2437R 2437L 2480R 2480L 2440R 2440L 2436R
amiR136-SCR amiR136-A2 low amiR136-A2 high
2451R 2451L 2458L 2437R 2437L 2480R 2480L 2440R 2440L 2436L
amiR136-SCR A2 high -A2 low amiR136-A2 high
2457R 2458L 2442L 2134R 2479R 2484R 2484L 2430R 2430L 2467R
amiR136-SCR amiR136-A2 low
2438L 2457R 2458L 2437R 2437L 2480R 2480L
amiR136-SCR amiR136-A2 high
2438L 2457R 2458L 2440R 2440L 2436R 2436L 2430R
amiR136-SCR amiR136-A2 high
2438L 2457R 2458L 2436L 2436L 2484R 2430R 2186R
amiR136-SCR amiR136-A2 low
2457R 2458L 2146L 2467R 2479R 2134L 2479L 2437R 2443R 2480R
amiR136-SCR amiR136-A2 high
2457R 2458L 2146L 2435R 2435L 2460R 2460L 2442R 2442L 2186R
amiR136-SCR amiR136-A2 high
2438L 2457R 2458L 2186R 2484R 2484L 2436R 2436L 2430R 2430L

Supplemental Figure S5. Western blots used for quantification of HTT suppression in the striatum 20 weeks post injection with (**A**) AAV5-shA2 and (**B**) amiR136-A2. L- left hemisphere, R – right hemisphere

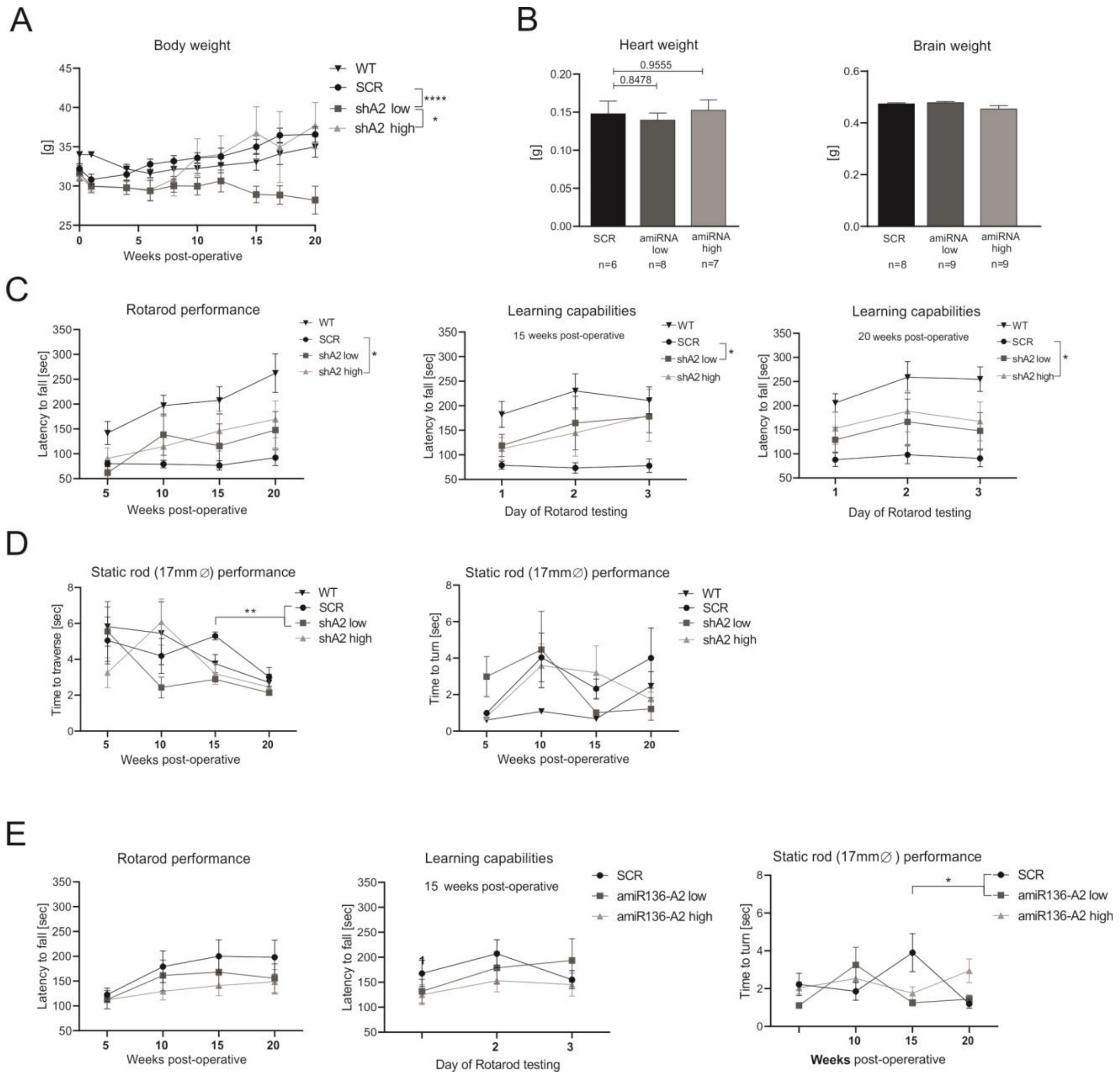

Supplemental Figure S6. The effects of shA2 and amiR136-A2 administration on body and organ weight and on behavior in YAC128 mice. (**A**) Body weight was measured twice at 5 week intervals throughout the experimental period and showed significant differences between the SCR- and shRNA-treated groups at both the low and high doses. WT animals were used as healthy controls. (**B**) The heart weight and brain weight did not change after amiR136-A2 treatment (**C**) When tested for 3 consecutive days, mice showed improvement in learning on the rotarod test toward the performance of healthy animals 15 weeks after injection with the low dose of shRNA (**D**) The results of the static rod test. Mice treated with shA2 traversed the 17 mm rod more quickly than WT mice and exhibited a significant performance difference compared to shSCR control-treated animals 15 weeks after treatment. There was no significant difference in time to turn parameter (**E**) YAC128 mice treated with amiR136-A2 did not show any improvement in performance in either motor performance or learning capabilities on the rotarod test; mice treated with the low dose of amiRNA showed an improvement in time turn on the 17-mm rod 15 weeks after injection. P values are indicated by asterisks (* < 0.05; ** < 0.01; **** < 0.0001). For body weight 2-way ANOVA was employed and for the spleen weight 1-way ANOVA was used, both with the Tukey's test.

Supplemental Table S1. Oligonucleotides used for the generation of amiRNA constructs.

Pri-miRNA shuttle	Insert	Oligonucleotide sequences 5'->3'
451	A2	CCCAAGAAGCTCTCTGCTCAGCCTGTCACAACCTACTGACTGCCAGGGCACTTGGGAATGGCAAGGGCTGCTGCAGCTGCTGCTGCTGCAGCAGCTGCAGCAGATCTTGCTATACCCAAGAAACGTGCCAGGAAGAGAACTCAGGACCCTGAAGCAGACTACTGGAAGGG
	G4	CCCAAGAAGCTCTCTGCTCAGCCTGTCACAACCTACTGACTGCCAGGGCACTTGGGAATGGCAAGGGCTGCTGCGGCTGCGGCTGCTGCCGCAGCCGCAGCAGATCTTGCCATTCCCAAGAAACGTGCCAGGAAGAGAACTCAGGACCCTGAAGCAGACTACTGGAAGGG
136	A2	CACTCCACTGCCCGACGTCGCCTCGGTGGTGTTGGATGAGCCCTCGGAGGGCTGCTGCAGCTGCTGCTGCTCGATTCTTATGCTCGAGCAGCAGCAGCTGCAGCAGTTCAGAGGGTTCTATCATTTCGTCGGATGGAAAGGAGTGTATTCTGAAGAT
	G4	CACTCCACTGCCCGACGTCGCCTCGGTGGTGTTGGATGAGCCCTCGGAGGGCTGCTGCGGCTGCGGCTGCTCGATTCTTATGCTCGAGCAGCcGCAGCcGCAGCAGTTCAGAGGGTTCTATCATTTCGTCGGATGGAAAGGAGTGTATTCTGAAGAT
122	A2	GACAATGGTGGAATGTGGAGGTGAAGTTAACACCTTCGTGGCTACACCTTAGCAGAGCTGGCTGCTGCAGCTGCTGCTGCTTGTCTAAACTATAGCAGCAGCAGCTGCAGCAGCCAGCTACTGCTAGGCTGTCTTGGCATCGTTTGCTTTGAGCAAGAAGGTTCATCT
	G4	GACAATGGTGGAATGTGGAGGTGAAGTTAACACCTTCGTGGCTACACCTTAGCAGAGCTGGCTGCTGCGGCTGCGGCTGCTTGTCTAAACTATAGCAGCCGCAGCCGCAGCAGCCAGCTACTGCTAGGCTGTCTTGGCATCGTTTGCTTTGAGCAAGAAGGTTCATCT
155	A2	GCCTGGAGGCTTGCTTTGGGCTGTATGCTGGCTGCTGCAGCTGCTGCTGCTGTTTTGGCCACTGACTGACAGCAGCAGCAGCTGCAGCAGCCAGGACACAAGGCCCTTTATCAGCACTCACATGGAACAAATGGCCC
	G4	GCCTGGAGGCTTGCTTTGGGCTGTATGCTGGCTGCTGCGGCTGCGGCTGCTGTTTTGGCCACTGACTGACAGCAGCcGCAGCcGCAGCAGTCAGGACACAAGGCCCTTTATCAGCACTCACATGGAACAAATGGCCC

Supplemental Table S2. Off-targets with full complementarity to the A2 insert.

Gene ID	Gene name	Localization	Expression in brain	Expression in other tissues
Golga4	Golgi autoantigen, golgin subfamily a, 4	ORF, 3' UTR*	Low**	High
Soga3	SOGA family member 3	ORF	High	Low
Maml1	Mastermind like transcriptional coactivator 1	ORF	Low	Low
Ccdc177	Coiled-coil domain containing 177	ORF	High	Low
Th	Tyrosine hydroxylase	ORF	High	Low
Ppp1r3f	Protein phosphatase 1, regulatory subunit 3F	ORF	Low	Low

* 3'UTR in transcript ENSMUST00000212593.1. ** based on publication Guo S. et al., DOI: 10.1016/j.bbrc.2020.05.170

Supplemental Table S3. Sequences of primers used for RT-qPCR and genotyping.

Gene	Primer orientation	Sequence
β-actin	F	AGAGCTACGAGCTGCCTGAC
	R	AGCACTGTGTTGGCGTACAG
Cccdc177	F	TCGGACAGGTAGAAAGAGCCAC
	R	CTGTTCCTGGCGGAAGCTCGA
Cnr1	F	ATCGGAGTCACCAGTGTGCTGT
	R	CCTTGCCATCTTCTGAGGTGTG
Darpp32	F	TCTCAGAGCACTCCTCACCAGA
	R	CACTCAAGTTGCTAATGGTCTGC
Drd2	F	CCTGTCCTTCACCATCTCTTGC
	R	TAGACCAGCAGGGTGACGATGA
Gfap	F	CACCTACAGGAAATTGCTGGAGG
	R	CCACGATGTTCCTCTTGAGGT
Golga4	F	GCAAATGGACCAGCAAGCAA
	R	GGGTTTTAGCGGAAGTCCCA
HTT	F	GTGCAGTGATGACGCAGAGT
	R	TCTTCGGGTCTCTTGCTTGT
Htt (for genotyping)	F	CCGCTCAGGTTCTGCTTTTA
	R	TGGACAGGGAACAGTGTTGG
HTT (for genotyping)	F	CCGCTCAGGTTCTGCTTTTA
	R	GGCTGAGGAAGCTGAGGAG
Iba1	F	TCTGCCGTCCAAACTTGAAGCC
	R	CTCTTCAGCTCTAGGTGGGTCT
Maml1	F	TCACAAGCAAGATGATGAGCACAG
	R	GCACGGAAGTCACTCCAGCA
Ppp1r3f	F	CCTGATGTTCGAGAGTCACTAGG
	R	TGCTGGTCAACATAACTTCGGGC
Soga3	F	AGATGGAGAAGCTGAGGGAAGAG
	R	AGTTGACAGGCATCCTCCTCGA
Th	F	GCCAAGGACAAGCTCAGGAA
	R	CTCAGTGCTTGGGTCAGGGT

Supplemental Table S4. Antibodies used for Western blot analysis and immunohistochemistry.

Protein	Dilution	Supplier	Secondary antibody
HTT (total)	1:2000 in milk 5% PBS-T	Abcam (ab109115)	R-POX Jackson, ImmunoResearch 1:1000, milk 5% PBS-T
polyQ	1:1000 in milk 5% PBS-T	Sigma-Aldrich (P1874)	M-POX Jackson, ImmunoResearch 1:1000, milk 5% PBS-T
plectin	1:1000 in milk 5% PBS-T	Cell Signaling (#12254)	R-POX Jackson, ImmunoResearch 1:1000, milk 5% TBS-T
calnexin	1:2000 in milk 5% PBS-T	Sigma-Aldrich (C4731)	R-POX Jackson, ImmunoResearch 1:1000, milk 5% PBS-T
HCN1	1:500 in milk 5% PBS-T	Abcam (ab176304)	R-POX Jackson, ImmunoResearch 1:1000, milk 5% PBS-T
RBM33	1:1000 in milk 5% PBS-T	Bethyl Laboratories (A303-926A)	R-POX Jackson, ImmunoResearch 1:1000, milk 5% PBS-T
HTT (aggregates)	1:50 in 4% NGS PBS-T	Sigma-Aldrich (**MAB5374**)	Anti-mouse, Vector Laboratories (MP-7802)
NeuN	1:500 in 4% NGS PBS-T	Millipore (MAB377)	Anti-mouse, Thermo Scientific, 1:1000, 4% NGS TBS-T
IBA1	1:1000 in 4% NGS PBS-T	WAKO (019-19741)	Anti-rabbit, Thermo Scientific, 1:1000, 4% NGS TBS-T
GFAP	1:400 in 4% NGS PBS-T	Millipore (MAB3402)	Anti-mouse, Thermo Scientific, 1:1000, 4% NGS TBS-T
DARPP-32	1:500 in 4% NGS PBS-T	R&D Systems (MAB4230)	Anti-rat, Thermo Scientific, 1:1000, 4% NGS TBS-T
