# Supplementary material for: A CAG repeat-targeting artificial miRNA lowers the mutant huntingtin level in the YAC128 model of Huntington's disease
Source: Mol Ther Nucleic Acids. 2022 May 5;28:702–15. doi: 10.1016/j.omtn.2022.04.031 (PMC9126840; doi:10.1016/j.omtn.2022.04.031)
Supplement: Document S2. Article plus supplemental information [file mmc2.pdf]

Molecular Therapy
Nucleic Acids
Original Article

# A CAG repeat-targeting artificial miRNA lowers the mutant huntingtin level in the YAC128 model of Huntington's disease

Anna Kotowska-Zimmer,[1] Lukasz Przybyl,[2] Marianna Pewinska,[1] Joanna Suszynska-Zajczyk,[3] Dorota Wronka,[2] Maciej Figiel,[4] and Marta Olejniczak[1]

[1]Department of Genome Engineering, Institute of Bioorganic Chemistry, Polish Academy of Sciences, Noskowskiego 12/14, 61-704 Poznan, Poland; [2]Laboratory of Mammalian Model Organisms, Institute of Bioorganic Chemistry, Polish Academy of Sciences, Noskowskiego 12/14, 61-704 Poznan, Poland; [3]Department of Biochemistry and Biotechnology, Poznan University of Life Sciences, 60-632 Poznan, Poland; [4]Department of Molecular Neurobiology, Institute of Bioorganic Chemistry, Polish Academy of Sciences, Noskowskiego 12/14, 61-704 Poznan, Poland

**Among the many proposed therapeutic strategies for Huntington's disease (HD), allele-selective therapies are the most desirable but also the most challenging. RNA interference (RNAi) tools that target CAG repeats selectively reduce the mutant huntingtin level in cellular models of HD. The purpose of this study was to test the efficacy, selectivity, and safety of two vector-based RNAi triggers in an animal model of HD. CAG repeat-targeting short hairpin RNA (shRNA) and artificial miRNA (amiRNA) were delivered to the brains of YAC128 mice via intrastriatal injection of AAV5 vectors. Molecular tests demonstrated that both the shRNA and amiRNA reduced the mutant huntingtin level by 50% without influencing endogenous mouse huntingtin. In addition, a concentration-dependent reduction in HTT aggregates in the striatum was observed. In contrast to the shRNA, the amiRNA was well tolerated and did not show signs of toxicity during the course of the experiment up to 20 weeks post injection. Interestingly, amiRNA treatment reduced the spleen weight to values characteristic of healthy (WT) mice and improved motor performance on the static rod test. These preclinical data demonstrate that the CAG-targeting strategy and amiRNA could make an original and valuable contribution to currently used therapeutic approaches for HD.**

## INTRODUCTION

Huntington disease (HD) is an inherited neurodegenerative disorder caused by the expansion of CAG repeats that encode a polyglutamine (polyQ) tract in the huntingtin protein (HTT). The underlying mutation is located in exon 1 of the 67-exon huntingtin (*HTT*) gene, and the presence of 36 or more CAG repeats is considered pathological.[1] A higher number of repeats results in earlier onset, faster progression, and increased severity of disease symptoms, with >60 CAG repeats leading to the juvenile form of HD.[2] Somatic CAG repeat instability further expands the CAG/polyQ tract and may serve as an important factor contributing to the selective vulnerability of brain tissues (e.g., the striatum and cortex) and cells (striatal medium spiny neurons) to HD.[3,4] Normal huntingtin is widely expressed and is essential for early embryogenesis and the development of the central nervous system.[5–7] Mutant HTT acquires a toxic function and forms intracellular aggregates that are linked to neuronal dysfunction and degeneration.[3,8,9] In addition, aberrant splicing of mutant HTT mRNA results in the production of the highly pathogenic exon 1 HTT protein.[10,11]

Therapeutics that lower the mutant HTT level, such as antisense oligonucleotides (ASOs), RNA interference (RNAi) tools, zinc finger transcriptional repressors, or small molecule inhibitors have shown promising results in preclinical studies.[12] Nonselective approaches that target both mutant and normal HTT are much more prevalent than allele-selective strategies based on single nucleotide polymorphisms (SNPs) or CAG tract length. However, a growing body of evidence suggests that huntingtin plays important functions in the adult brain; thus, selective approaches are much safer.[13] In 2021, three clinical trials of HTT-lowering ASOs, including allele-selective (NCT03225833 and NCT03225846) and nonselective (NCT03761849) ASOs, were terminated. Of special importance for the HD community is the discontinuation of a phase III study of tominersen (nonselective), which had been demonstrated to be safe, well tolerated, and efficient in reducing the HTT level in the cerebrospinal fluid during a previous phase I/IIa trial.[14] Wave Life Sciences trials (allele-selective) were terminated due to the lack of significant change in the level of the mutant HTT in trial participants treated with WVE-120101 and WVE-120102, compared with those treated with placebo. In the case of Roche, patient groups treated with the tominersen were gradually but clearly starting to do worse than people in the group treated with a placebo. Therefore, there is a need for the development

Received 11 September 2021; accepted 29 April 2022;
https://doi.org/10.1016/j.omtn.2022.04.031.

**Correspondence:** Marta Olejniczak, Department of Genome Engineering, Institute of Bioorganic Chemistry, Polish Academy of Sciences, Noskowskiego 12/14, 61-704 Poznan, Poland.
**E-mail:** marta.olejniczak@ibch.poznan.pl

 © 2022 The Author(s).
This is an open access article under the CC BY license (http://creativecommons.org/licenses/by/4.0/).

of new therapeutic strategies for HD and their proper validation in preclinical studies.

RNAi technology uses exogenous small interfering RNA (siRNA) and cellular proteins (the RNA-induced silencing complex, RISC) for selective degradation of target transcripts. Chemical and structural modifications of siRNA (divalent siRNA) allowed potent and persistent silencing of huntingtin in the brains of HD mice, which lasted for at least 6 months.[15] Longer silencing effects can be achieved by vector-based RNAi triggers, such as artificial microRNAs (amiRNAs) and short hairpin RNAs (shRNAs).[16] These molecules resemble miRNA precursors (pri-miRNAs and pre-miRNAs, respectively) and undergo intracellular processing by the endonucleases Drosha and/or Dicer to form mature siRNAs. An amiRNA based on pri-miR-451 and delivered via an AAV5 vector (AMT-130) was demonstrated to be safe and efficient for allele-nonselective silencing of huntingtin in a few animal models of HD.[17–19] A phase I/IIa clinical trial (NCT0412049) was started this year to investigate the safety and persistence of AMT-130 in the brain. In other approaches, pri-miR-30- and pri-miR-155-based amiRNAs were used to lower the HTT level using AAV2/1 and AAV9 vectors, respectively.[20–22] However, all the above examples are nonselective approaches that target both mutant and normal HTT.

In our previous studies, we demonstrated that shRNAs targeting CAG repeats are selective for mutant huntingtin in cellular models of HD.[23,24] Allele selectivity was achieved by the introduction of mismatches to the siRNA:target duplexes, which changed the mechanism of action from transcript degradation (siRNA-like) to translation inhibition (miRNA-like).[25,26] In addition, the same shRNAs were efficient in selective inhibition of mutant proteins in other polyQ models,[24] thus supporting the idea of using universal CAG-targeting therapeutics for the treatment of polyQ diseases.

Here, we designed and characterized a CAG-targeting amiRNA vector based on a novel pri-miR-136 backbone. Then, we compared the efficacy, selectivity, and safety of the most universal CAG-targeting shRNA (shA2) and corresponding amiRNA in an animal model of HD. RNAi tools were delivered to the brains of YAC128 mice via intrastriatal injection of AAV5 vectors. Regarding the two molecules tested, the amiRNA was efficient, showed allelic preference for the mutant HTT, and was well tolerated for up to 20 weeks post injection. It reduced the number of polyQ aggregates in the striatum, a major hallmark of HD. This preclinical study is an important step in the clinical translation of the RNAi-based CAG-targeting strategy.

## RESULTS

### Design and characteristics of the CAG repeat-targeting artificial miRNA

The most efficient and allele-selective shRNAs targeting the CAG tract were selected from our previous study[24] and used to design more complex amiRNA molecules composed of a siRNA insert and a pri-miRNA scaffold. siRNA inserts contain a single A (A2) and a double G (G4) interruption within a CUG sequence, which generate A:A and G:A mismatches with a target sequence in the transcript (Figure 1A). CAG-targeting siRNAs were embedded within four naturally occurring pri-miRNA backbones: human pri-miR-451, pri-miR-122, and pri-miR-136 and mouse pri-miR-155 (Figure 1A). The cellular processing of these pri-miRNAs is well characterized.[20,27–29] Based on miRBase (http://www.mirbase.org/)[30] analysis and our previous study,[28] these pri-miRNAs show high guide-to-passenger strand ratios. In addition, pri-miR-451 undergoes noncanonical, Dicer-independent processing, which does not result in the formation of a passenger strand.[31] In the amiRNAs based on pri-miR-155 and pri-miR-451, the stem contains the bulges that exist in naturally occurring pri-miRNAs to improve their processing, while the amiRNAs based on shmiR-136 and shmiR-122 exhibit full base complementarity within the hairpin stem (Figure 1A). amiRNA expression cassettes driven by the cytomegalovirus (CMV) promoter were inserted upstream of the copepod GFP (copGFP) reporter gene expressed under the control of the EF-1$\alpha$ promoter. Lentiviral vectors encoding amiRNAs and control vectors were generated.

In the first step, we analyzed the silencing efficiency of the amiRNAs in a cellular model of HD. Patient-derived fibroblasts (GM04281; 17/68 CAG repeats) were transduced with lentiviral particles at an MOI of 10, and the HTT protein level was analyzed by western blotting. The most effective reagents (amiR136-A2 and amiR451-A2) decreased the mutant HTT protein level by approximately 50%, leaving normal huntingtin level unchanged (Figure 1B). The less effective amiRNAs based on pri-miR-122 and pri-miR-155 caused a 20% to 40% reduction in the mutant HTT level. They also showed worse selectivity. The most allele-selective construct based on pri-miR-136 was chosen for further analysis as a potential candidate for HD therapy. Then amiR136-A2 was tested in other HD patient-derived cell line containing shorter CAG repeat tract in the mutant *HTT* allele (GM04869; 15/47 CAG repeats) (Figure 1C). With the use of two anti-HTT antibodies, we demonstrated that amiR136-A2 significantly decreased the mutant HTT protein level. However, the allele-discriminating properties of the tested reagent were lower than those observed for longer mutant *HTT* alleles. The allele-selective potential of amiR136-A2 was analyzed by a luciferase assay using *HTT* exon 1 containing 16, 40, and 57 CAG repeats as targets (Figure 1D). We observed a repeat length-dependent silencing by amiR136-A2. An HTT silencing efficiency of approximately 50% was achieved for the target sequence with 40 CAG repeats, which is one of the shortest mutant variants observed in patients. Given that the expression of the normal *HTT* variant with 16 CAG repeats was reduced by approximately 20%, this result confirmed the allelic preference of amiR136-A2.

To better characterize this molecule, we analyzed the products of amiR136-A2 processing by Drosha and Dicer. HEK293T cells were transfected with plasmids encoding amiR136-A2, and small RNA-sequencing analysis was performed. The reagent exhibited predominance of the guide siRNA strand originating from the 5′ arm, reaching more than 80% of the reads (Figure 1E). Nearly 70% of the molecules contained an A substitution at position 8 relative to

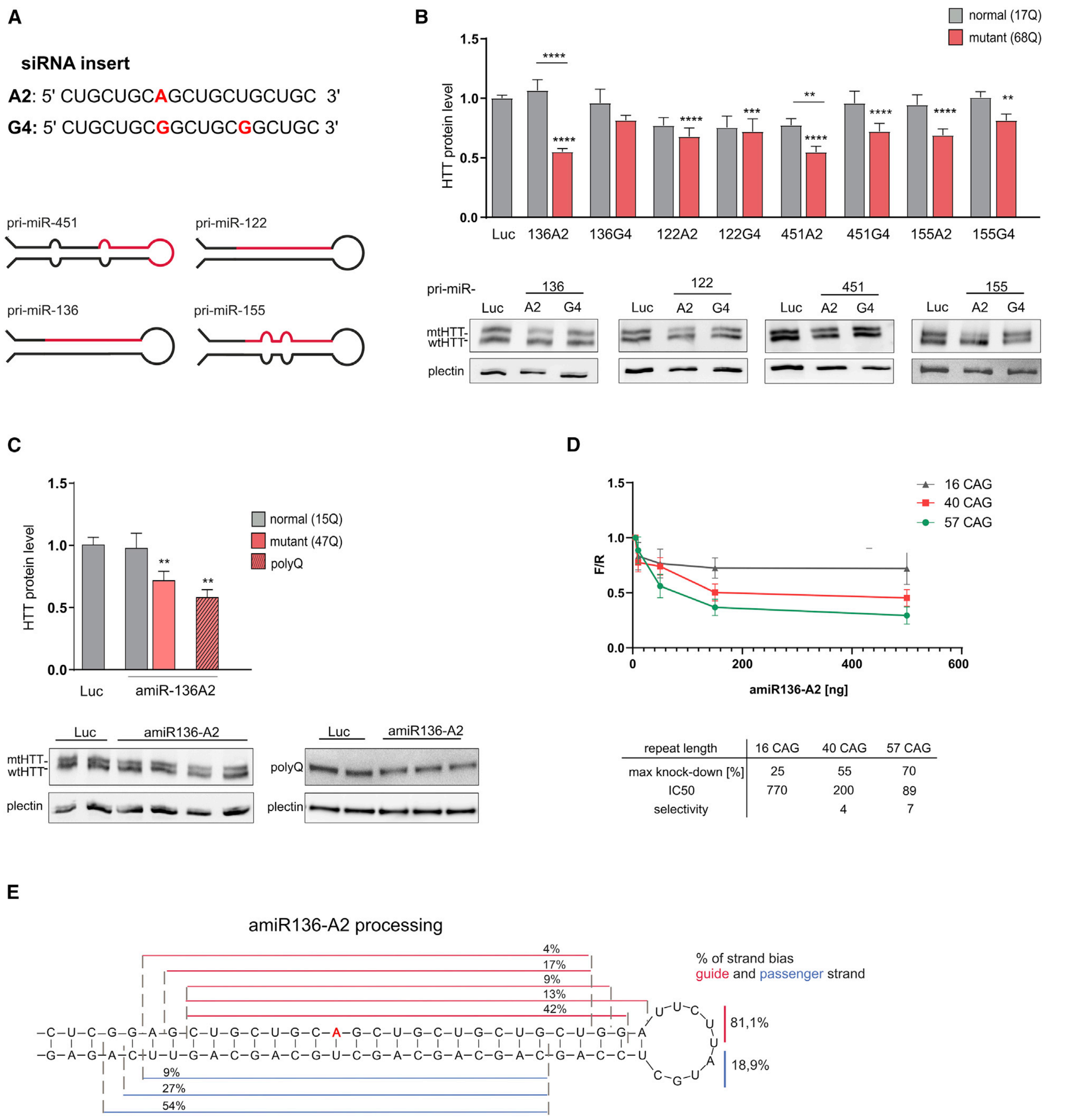


**Figure 1. *In vitro* analysis of the efficiency and allele selectivity of different amiRNAs**

(A) Schematic representation the siRNA inserts and the pri-miRNA shuttles used to construct the amiRNAs. (B) Western blot analysis of HTT levels in HD patient-derived fibroblasts (cell line GM04281, 17/68Q) 7 days posttransduction with lentiviral particles (MOI of 10), containing expression cassettes with amiRNAs based on the pri-miR-136, pri-miR-122, pri-miR-155, and pri-miR-451 shuttles. Signal intensities of the protein bands were normalized to those of plectin and compared using a one-sample t test. The bars on the graph indicate the mean protein levels ± SEMs (from at least three biological and technical replicates, n = 9). p values are indicated by asterisks (*$p < 0.03$, **$p < 0.002$, ***$p < 0.0002$, ****$p < 0.0001$). (C) Western blot analysis of HTT levels in HD patient-derived fibroblasts (cell line GM04869, 15/47Q) 7 days posttransduction with lentiviral particles (MOI of 10), containing expression cassette with amiR136-A2. Signal intensities of the protein level were normalized to plectin protein levels and compared using a one-sample t test. The graph bars represent the mean value of protein levels ± SEM (from at least three biological and technical replicates, n = 9). p values are

*(legend continued on next page)*

the 5′ end, and the predominant length of the product was 22 nt. A similar pattern of processing was also observed for the shA2 molecule in our previous studies[23,24]; however, the number of reads representing mature siRNA molecules for the amiRNA was approximately 10 times lower than that for the shRNA and was comparable to the endogenous miRNA level (Figure S1).

### ShA2 and amiR136-A2 reduce the mutant HTT level *in vivo* in an allele-selective manner

Two types of vector-based RNAi tools—shRNAs and amiRNAs—can be used to achieve long-lasting silencing effects *in vivo*. Therefore, to directly compare the efficiency and allele selectivity of the CAG-targeting RNAi triggers, shA2 and amiR136-A2 constructs were generated in AAV5 vectors for direct delivery to the striatum of mice. The shRNA and amiRNA were expressed under the control of the Pol III promoter (H1 promoter) and Pol II promoter (a CAG promoter consisting of the cytomegalovirus immediate-early enhancer fused to the chicken β-actin promoter), respectively (Figure 2A). Transgenic YAC128 mice, which express full-length human HTT with 125 CAG repeats interrupted by nine CAA repeats, were used as the HD model[32] (Figure 2B). The CAA CAA CAG CAA interruptions are located at repeats 24–28, 109–113, and a single CAA triplet is located at repeat 124. This structure of interruptions still leaves 80 pure CAG repeats and allows the study of CAG repeat-targeting strategies. The presence of mouse Htt (mHtt) with 7 CAG repeats allows indirect analysis of allele selectivity in this model. It is worth noting that the expression level of human HTT is approximately 75% that of endogenous mHtt.[33]

To investigate the distribution of AAV5 in the brains of YAC128 mice, we injected AAV5-GFP unilaterally into the striatum at three doses: $1 \times 10^9$, $1 \times 10^{10}$, and $1 \times 10^{11}$ gc/animal (n = 3 mice per dose). One month post injection, mice were killed, and coronal and sagittal sections of their brains were prepared for fluorescence microscopy. For the highest concentration of AAV5-GFP, we observed widespread distribution of vector at the injection site and in surrounding regions. The GFP signal was observed in the striatum as well as in the hippocampus. Deeper layers of the cortex were also transduced (Figure 2C).

In the next step, AAV5 vectors expressing shA2 or amiR136-A2 were injected unilaterally into the striatum of 16- or 12-week-old mice, respectively (n = 10 mice per vector). Scramble shRNA (shSCR) and amiRNA (amiR136-SCR) were used as controls. GFP was excluded from the expression cassettes to eliminate the risk of inducing the host immune response. Mice received $1 \times 10^{11}$ gc of AAV5-shRNA or $3 \times 10^{11}$ gc of AAV5-amiRNA. One month post injection, mice were killed, and their brains were processed to assess the silencing efficiency and the presence of vector DNA. To quantify AAV5 genome copies in the striatum, hippocampus and cortex we performed RT-qPCR with primers specific for the H1 or CAG promoter, depending on the construct. The vector DNA levels in homogenates from injected mice correlated with the silencing efficiency of the HTT protein (Figure 2D). Generally, animals that showed the strongest vector transduction (more than $1 \times 10^{11}$ vector genome copies per microgram of DNA) showed the greatest reduction in the HTT protein level relative to the SCR control-treated group. A similar average silencing efficiency was observed for shA2 and amiR136-A2 in the striatum (~30%). We also analyzed each sample individually, and the maximum efficiency of HTT silencing in the striatum was ~65% and ~50% for shA2 and amiR136-A2, respectively. The endogenous mHtt level was not significantly reduced. In the other brain regions, the silencing of HTT was also noticeable. In the hippocampus, the silencing efficiency was similar to that in the striatum, possibly due to the proximity of these two regions, and was 30% and 20% for shA2 and amiR136-A2, respectively. The smallest effect was observed in the cortex; a silencing efficiency of 20% was achieved only by amiR136-A2 (Figure S2A). In addition, in some animals, we observed greater HTT silencing in the hippocampus and cortex than in the striatum (Figure S2B). The high variability of HTT silencing between individuals can be partially explained by the uneven distribution of AAV5 (Figure S3).

### Efficacy comparison of shA2 and amiR136-A2 in a long-term experiment

To evaluate the efficacy of HTT silencing over an extended period, adult mice (12–14 weeks old) received bilateral intrastriatal injections of AAV5 carrying shA2 or amiR136-A2 at low and high doses (n = 10 mice). Every 5 weeks post injection, the body weights of the mice were evaluated, and behavioral tests were performed (Figure 3A). Twenty weeks post injection, the mice were killed, and the brains, hearts, and spleens were removed, weighed, and snap frozen for further molecular analysis. DNA, protein, and RNA were isolated from the striatum, hippocampus, and cortex for analysis of vector genome copies, analysis of protein levels by western blotting, and analysis of transcript levels by RT-qPCR, respectively. Two animals from each group were also subjected to perfusion for further immunohistochemical analysis of brain tissues.

Western blot analysis revealed that the HTT protein level in the striatum was significantly reduced by 50% in both the low- and high-dose shA2-treated groups compared with the SCR control-treated group (Figure 3B). Similar silencing efficiencies were achieved using amiR136-A2 at the high dose (~45% and ~50% using a polyQ-specific antibody) (Figure 3C). The low dose of amiR136-A2 caused a decrease of 30% in the HTT level. Both molecules silenced the

indicated by asterisks (**$p < 0.002$). (D) The graph shows the results of Luc reporter knockdown by the amiR136-A2 construct. HEK293T cells were co-transfected with 50 ng of Luc reporters and 5, 10, 50, 150, or 500 ng of amiRNA constructs. The maximal knockdown efficiency achieved with amiR136-A2 (%); the half-maximal inhibitory concentration (IC50) and allele selectivity are shown in the table. (E) Next-generation sequencing analysis of the amiR136-A2 processing pattern in HEK293T cells. The guide strand is indicated in red, and the passenger strand is indicated in blue. Cleavage sites are shown on both strands corresponding to the length of released siRNA variants.

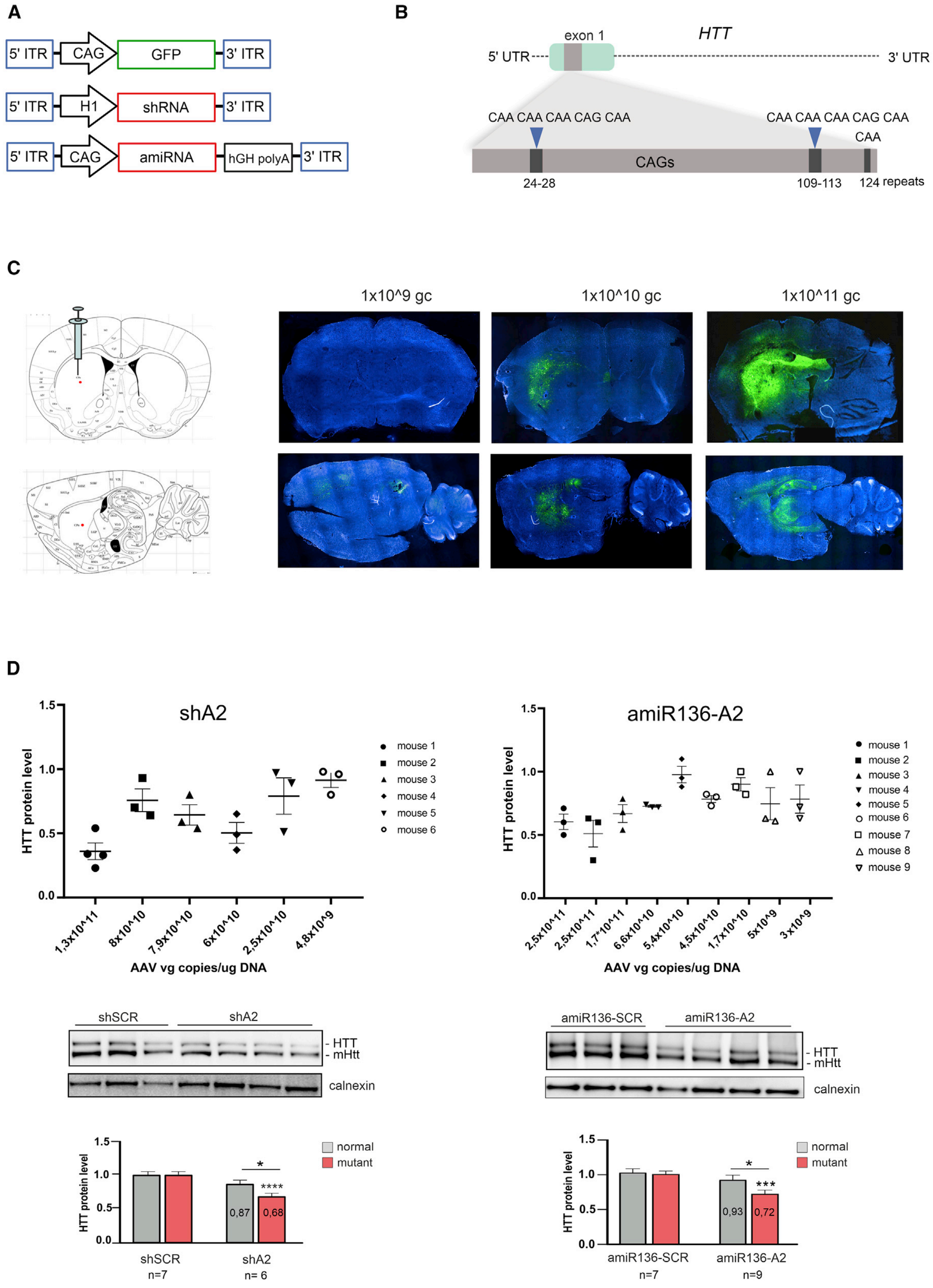


(legend on next page)

expression of HTT in an allele-selective manner, and there was no statistically significant silencing of mHtt with a normal-length-CAG tract. Allele-selective silencing of HTT by shA2 was also observed in the hippocampus and cortex, with silencing efficiencies of approximately 30% and 20%, respectively (Figure S4A). Interestingly, amiR136-A2 reduced the HTT level in the hippocampus by 45% at the low dose and by 35% at the high dose (Figure S4B). Similar to the observations in the short-term experiment, we observed interindividual variability in HTT silencing, and a difference was also observed between brain hemispheres (Figure S5). Analysis of the *HTT* transcript level did not reveal any differences between control- and amiR136-A2-treated animals, confirming translation inhibition mechanism of action (Figure 3D). YAC128 mice exhibit age-dependent neuropathology manifested as whole brain atrophy including striatal loss and the presence of HTT aggregates. We observed a dose-dependent reduction in the number of polyQ aggregates in the striata of amiR136-A2-treated mice (Figure 4).

### amiR136-A2 is well tolerated for up to 20 weeks post injection

It has been previously demonstrated that shRNA vectors can be toxic *in vivo*.[34–36] During the course of the experiment, we observed abnormal behavior of animals treated with AAV5-shRNAs, and some of them had to be killed before termination of the study, specifically, five mice treated with shA2 at the high dose, 3 mice treated with shA2 at the low dose, and one mouse treated with shSCR (control). Consequently, the number of animals per group decreased over time, and some analyses could not be performed. In contrast, amiRNA treatment did not induce any abnormalities, and no signs of toxicity were observed.

YAC128 mice exhibit a characteristic body weight increase starting at the age of 2 months.[37] Interestingly, mice injected with the low dose of shA2 weighed less and did not gain weight over time, in contrast to mice in all other groups (Figure S6A). There was no difference in body weight among wild-type (WT), amiR136-SCR-treated, and amiR136-A2-treated animals (Figure 5A).

Expression of mutant huntingtin has been previously shown to increase organ weight.[37] Therefore, after the experiment, hearts, brains, and spleens were weighed. Significant differences in organ weights between amiR136-SCR control- and amiR136-A2-treated animals were found only for the spleen (Figures 5A and S6B). Both groups treated with amiR136-A2 showed significantly smaller spleens (SCR = 0.135 g versus 0.105 g versus 0.102 g), resembling spleen weights characteristic of healthy (WT) mice (0.106 g ± 0.002).[37]

To evaluate whether injection of AAV5-amiR136-A2 induces neuroinflammation, striatal tissue sections were stained with antibodies against Iba-1 (a marker of microglia), GFAP (a marker of astrocytes), NeuN (a marker of neurons), and DARPP-32 (a marker of medium spiny neurons). We did not observe histopathologic changes in the injected brain regions (Figure 5B). These results were also confirmed by RT-qPCR analysis of the *Gfap* and *Iba-1* transcript levels, which were similar to those in SCR control-treated mice (Figure 5C).

We evaluated the selectivity of amiR136-A2 for mutant HTT by analyzing the levels of proteins encoded by other genes containing long CAG tracts, including *Rbm33* (10 CAG repeats) and *Hcn1* (>30 CAG repeats with a 4xCAA). Western blot analysis did not reveal any differences in these protein levels between SCR control-treated and amiR136-A2-treated animals (Figure 5D). Then, using bioinformatic analysis, we selected transcripts with full complementarity to A2 siRNA (Table S2). These transcripts included *Golga4*, *Soga3*, *Maml1*, *Ccdc177*, *Th*, and *Ppp1r3f*. Only one (*Golga4*) of the six tested transcripts was downregulated by amiR136-A2 at the high dose; however, this downregulation was statistically insignificant (Figure 5E). The human counterpart does not contain a sequence fully complementary to A2 siRNA.

### ShA2 and amiR136-A2 improve some motor and cognitive deficits

YAC128 mice exhibit progressive motor, cognitive, and psychiatric abnormalities.[38,38–41] During the course of the experiment, mice were subjected to an accelerated rotarod test to assess motor deficit improvement (5, 10, 15, and 20 weeks post injection). Throughout the experiment, mice treated with the high dose of shA2 performed significantly better than mice treated with control shSCR ($p = 0.0398$) (Figure S6C). Although mice injected with the low dose of shA2 performed similarly throughout the course of the experiment, the differences were not significant in comparison with performance in the shSCR group due to the smaller numbers of animals tested. In addition, these mice did not show significant differences compared with mice treated with the high dose. However, treatment with the low dose of shA2 significantly influenced the learning capabilities of YAC128 mice when the performance on each of the 3 days of testing was compared separately at 15 weeks after surgery ($p = 0.0444$). Mice that received the high dose of shA2 behaved similarly at that time point, and the improvement was not significant; however, 5 weeks later, the difference in performance was significant ($p = 0.0158$). In addition to the latency to fall, the distance, number of rotations to

**Figure 2. Analysis of the distribution of AAV5-GFP in the YAC128 mouse brain and analysis of the efficiency and allele selectivity of CAG-targeting RNAi triggers *in vivo* in a short-term experiment**

(A) Schematic representation of AAV5 vectors encoding GFP, shRNA, or amiRNA. (B) Representation of the YAC128 HTT transgene sequence and location of the CAA interruptions within the CAG tract. (C) Coronal and sagittal sections of mouse brains after intrastriatal injection of AAV5-GFP at three increasing doses: $1 \times 10^9$, $1 \times 10^{10}$, and $1 \times 10^{11}$ gc/mouse. (D) Analysis of HTT protein silencing and the number of AAV5 vector genome copies in the striatum of YAC128 mice. qPCR was used to quantify AAV5 genome copies in the brain structures of shA2-and amiR136-A2 injected mice (n = 6 and n = 9, respectively) 1 month post injection. Primers specific for the H1 and CAG promoters were used, and the gc values were calculated based on the standard curve. Western blots show examples of the results. Signal intensities of the protein bands were normalized to those of calnexin and compared using Student's t test. The bars on the graph indicate the mean protein levels ± SEMs (n = 6 for shRNA, n = 9 for amiRNA). p values are indicated by asterisks (*$p < 0.03$, **$p < 0.002$, ***$p < 0.0002$, ****$p < 0.0001$).

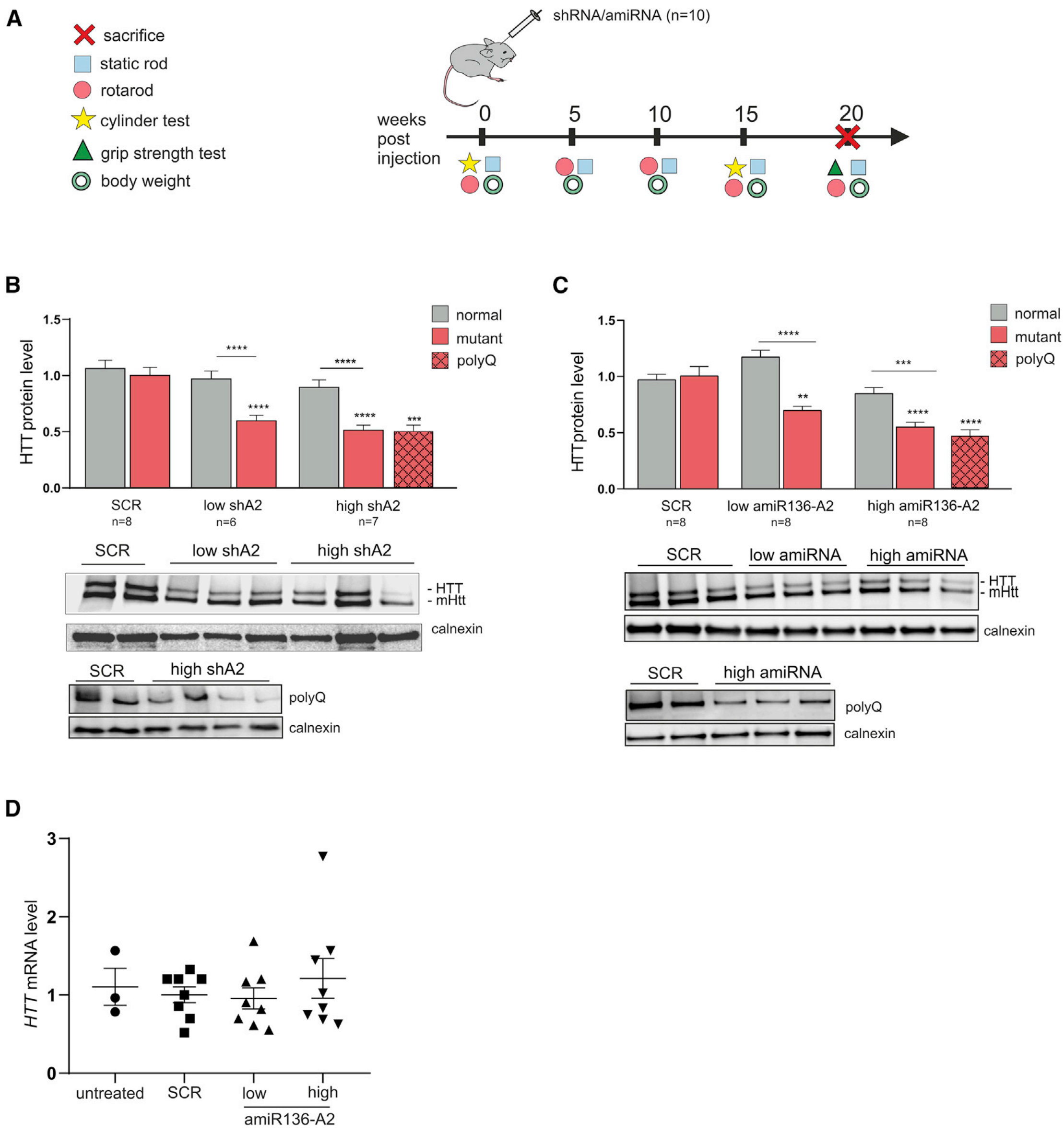


**Figure 3. Analysis of HTT protein and mRNA levels 20 weeks post intrastriatal injection of mice with A2 shRNA and amiRNA**

(A) Overview of the study design showing the timeline of AAV5-shA2 and amiR136-A2 injections, behavioral tests, and experimental endpoints. (B) Western blot analysis of the HTT level in the striatum after shA2 treatment. (C) Western blot analysis of the HTT level in the striatum after amiR136-A2 treatment. Signal intensities of the protein bands were normalized to those of calnexin and compared using Student's t test. Two antibodies were used to visualize HTT: one detecting both forms of protein, mutant and normal, and the second detecting only mutant protein (polyQ antibody). The bars on the graph indicate the mean protein levels ± SEMs (n = 8 for shSCR, 6 for shA2 low dose, 7 for shA2 high dose; 8 for amiR136-A2). p values are indicated by asterisks (*$p < 0.03$, **$p < 0.002$, ***$p < 0.0002$, ****$p < 0.0001$). (D) Analysis of the *HTT* mRNA level after amiR136-A2 treatment.

fall, and speed at falling were measured. All of the measurements showed similar results (data not shown).

To further assess the motor performance of mice, a static rod test was performed. Every 5 weeks, mice were placed facing outward on a rod of a certain diameter ranging from 28 to 10 mm, and the time the mouse needed to turn around and traverse the rod was measured. The most relevant rod has a diameter of 17 mm. Wider rods are easily traversed by mice, and a 10-mm rod is highly challenging for small rodents. Mice treated with shA2 traversed the 17 mm rod even more quickly than WT mice and exhibited a significant performance difference compared with shSCR control-treated animals 15 weeks after treatment (Figure S6D). The time to turn showed a similar trend, but the difference was not

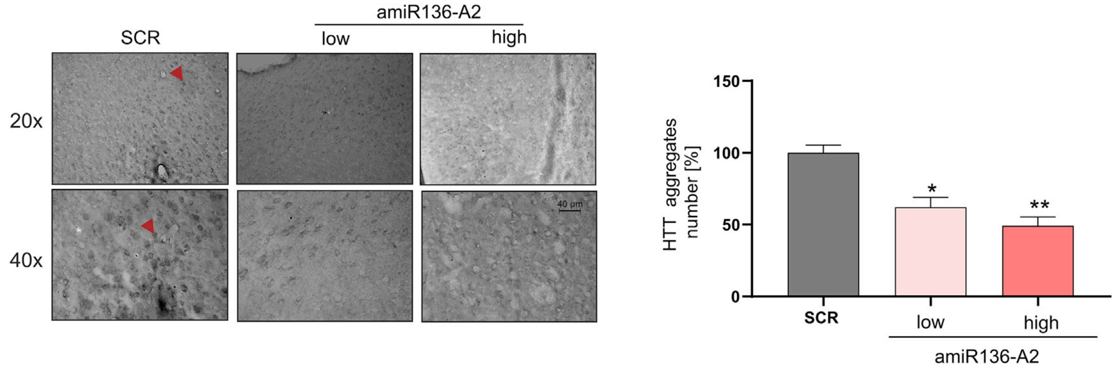


**Figure 4. Reduction of the number of HTT aggregates 20 weeks post injection with amiR136-A2**
Immunohistochemical (IHC) staining of the striatum using the EM48 antibody, which specifically reacts with intranuclear mutant HTT aggregates. Representative aggregates are indicated by the red arrow. The bars on the graph show the percentage reduction in HTT aggregates after amiR136-A2 treatment. The data were analyzed using one-way ANOVA. p values are indicated by asterisks (*$p < 0.03$, **$p < 0.002$). The bars on the graph show the mean protein levels ± SEMs (n = 2 for amiR136-SCR, 4 for amiR136-A2).

statistically significant. Similar trends were observed for other rod diameters and experimental time points.

Similar to shA2-treated animals, amiR136-A2-treated animals were evaluated using a battery of behavioral tests, including rotarod, static rod, grip strength, and cylinder tests. In the assessment of motor and learning capabilities using the rotarod test, the performance of mice treated with either amiR136-A2 concentration did not differ significantly compared with that of SCR control-treated mice. In contrast, on the static rod test, amiR136-A2-treated mice exhibited a significant improvement in the time to turn on the 17-mm diameter rod 15 weeks after treatment (3.9 versus 1.25 versus 1.76 s) (Figure S6E). Animals did not show any differences on the cylinder or grip strength tests (data not shown).

## DISCUSSION

Therapeutic strategies that lower the HTT level have been used in a number of preclinical studies and have demonstrated promising results in decreasing HD pathology.[14] However, apart from ZF transcription inhibitors,[42] there are no allele-selective approaches based on viral delivery and single administration of therapeutic agents.

Here, we developed a CAG-targeting amiRNA that efficiently and preferentially reduced the mutant HTT level in an animal model of HD. Because cellular biogenesis of vector-based RNAi triggers is difficult to predict, their selectivity with respect to the original siRNA can be reduced or lost.[43,44] Our previous efforts to find a pri-miRNA scaffold that generates homogeneous siRNA products,[28] and detailed analysis of pri-miR-136 processing allowed us to preserve the selectivity and efficacy of the amiR136-A2 vector. By direct comparison of the corresponding shRNA and amiRNA, we confirmed that shRNA can be toxic *in vivo*, probably due to saturation of the miRNA biogenesis pathway.[45] In contrast, amiR136-A2, which enters the miRNA biogenesis pathway at an early step and generates quantity of mature siRNA approximately 10 times lower than that generated by shA2, did not cause any overt symptoms of toxicity. However, both the shRNA and amiRNA variants reduced the mutant HTT level by ~50% 20 weeks post injection when administered at the high dose. This efficiency of HTT silencing was sufficient to observe a reduction in the number of polyQ aggregates in the striatum, improvements in some motor and learning deficits, and a reduction in the spleen weight to values characteristic of those in healthy mice. It has been postulated that mutant huntingtin expression increases organ weights (except those of the brain and testis), perhaps via a central mechanism originating in the brain.[33,37,39] This suggests the possibility that a reduction in the mutant HTT level in the brain may influence the spleen weight, but this hypothesis requires further investigation.

In general, CAG repeat-targeting strategies carry a risk of unintended targeting of other genes. Because of the miRNA-like translation inhibition mechanism, only transcripts containing long, uninterrupted CAG repeats can be efficiently silenced by amiR136-A2. In addition, transcripts with full complementarity to the A2 molecule (especially in the 3′UTR) can be degraded by a siRNA-like mechanism. Analysis of selected proteins and transcripts did not reveal any significant off-target effects. Since there is little similarity between the repeated sequences of mice and humans, further analyses in human neurons are necessary. The role of the mutant HTT transcript, which is not degraded by amiR136-A2, also requires further clarification. The toxicity of RNA containing long CAG repeats is mainly correlated to the production of the toxic forms of proteins.[10,11,46–48] Since our therapeutic agent blocks translation, we can assume that it prevents the formation of toxic proteins, and thus the negative effects mentioned in these studies.

Previous studies demonstrated that direct injection of AAV5 into the parenchyma ensures widespread distribution of the vector in the CNS and sufficient transduction of deep brain structures.[17,49,50] In addition, a recent study confirmed that amiRNAs are present in vesicles 2 years post injection into the brains of NHPs.[51] These results support the choice of AAV5 as a delivery vehicle for amiR136-A2; however, the high variability in the silencing efficiency between individuals

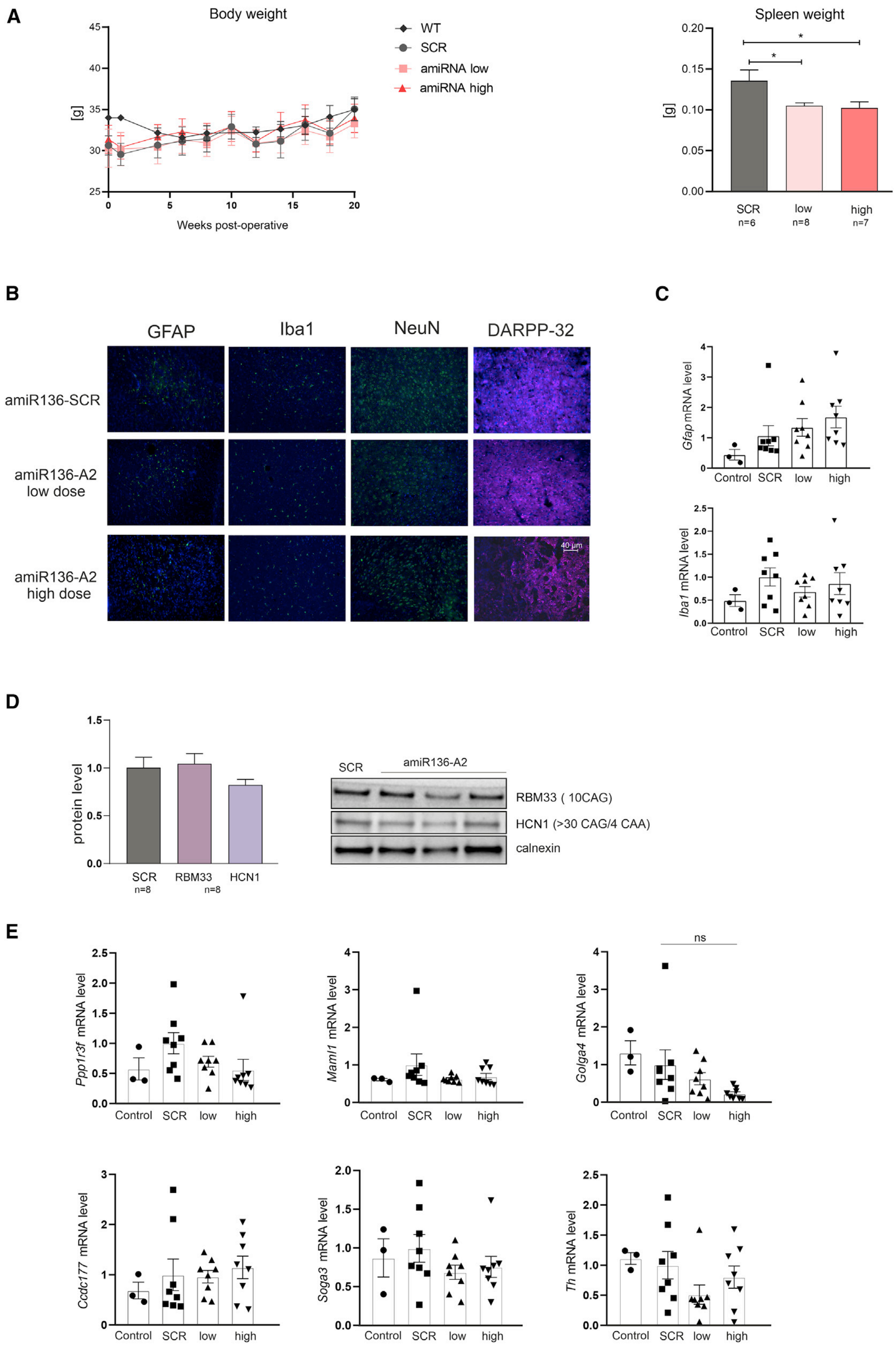


*(legend on next page)*

and the low silencing efficiency in the cortex suggest that the delivery and distribution of RNAi vectors in the brain should be improved. The uneven distribution of amiR136-A2 may be a cause of the weak improvements in motor deficits. YAC128 mice represent a "late-onset" model, and it is possible that examination at later time points (e.g., in $\geq$ 9-month-old animals) may reveal more behavioral improvements (our tested animals were 8 months old).

The most advanced RNAi-based approach using nonselective amiRNA and AAV5 vectors (AMT-130) is currently in a phase I/IIa clinical trial (NCT0412049). The main advantages of this approach compared with more advanced ASOs are the possibility of a single administration and the long-term effects. Our strategy gives an additional benefit of allele selectivity and possible universality for the treatment of other polyQ disorders. Overall, this preclinical study is an important step in the clinical translation of vector-based CAG repeat-targeting strategies.

## MATERIALS AND METHODS

### Cell culture

Fibroblasts from HD patients (GM04281 and GM04869) were obtained from Coriell Cell Repositories (Camden, NJ) and grown in minimal essential medium (MEM) (Sigma-Aldrich, St. Louis, MO) supplemented with 10% fetal bovine serum (FBS) (Sigma-Aldrich) and antibiotics (Sigma-Aldrich). HEK293T cells were grown in Dulbecco's modified Eagle's medium (DMEM) (Sigma-Aldrich) supplemented with 8% FBS, antibiotics, and L-glutamine (Sigma-Aldrich).

### Plasmids and viral vectors

For experiments performed in cell cultures, the amiRNA expression cassettes were generated from DNA oligonucleotides (Sigma-Aldrich, see the sequences in Table S1). Pairs of oligonucleotides were annealed and ligated into the pCDH-CMV-MCS-EF1-Puro (System Biosciences, Palo Alto, CA) expression plasmid and verified through sequencing. For lentivirus production, the plasmids were cotransfected with the packaging plasmids pPACKH1-GAG, pPACKH1-REV, and pVSVG (System Biosciences) into HEK293TN cells. The medium was collected on days 2 and 3, and the viral supernatants were passed through 0.45-μm filters and concentrated using PEGit Virus Precipitation Solution (System Biosciences). The lentiviral vectors were resuspended in Opti-MEM (GIBCO, Invitrogen, Carlsbad, CA), and the virus titers (TU/mL) were determined through flow cytometry (Accuri C6, BD Biosciences, San Jose, CA) based on copGFP expression. Transduction of fibroblasts was performed at MOI of 10 in the presence of polybrene (4 μg/mL). Total protein was harvested 7 days post transduction. The luciferase (Luc) construct was used as a negative control.

For *in vivo* experiments, the shRNA and amiRNA constructs were used for the production of the AAV5 vectors. The shRNAs were expressed under the control of the H1 Pol III promoter and they contained a 22-base pair stem and a 10-nt miR-23 loop; the amiRNAs were expressed under the control of the CAG Pol II promoter. We used shSCR (scramble) and amiR136-SCR constructs as negative controls for silencing. AAV5 vectors were produced in the HEK293 cell system by Vigene Biosciences (Rockville, MD).

### Luciferase assays

For luciferase assays, HEK293T cells were cultured in 24-well plates in DMEM supplemented with 10% FBS. The next day, the cells were cotransfected with two types of plasmids: constructs containing exon 1 of the *HTT* gene with defined numbers of CAG repeats (16, 40 and 57) with Renilla and firefly luciferase sequences,[24] and constructs containing amiR136-A2, using Lipofectamine 2000 (Invitrogen, Thermo Fisher Scientific, Carlsbad, CA). Cells were cotransfected with 50 ng of the HTT target reporter plasmid, and 5, 10, 50, 150, or 500 ng of the amiRNA construct. Forty-eight hours after transfection, cells were harvested and lysed using Passive Lysis Buffer (Promega, Madison, WI). The bioluminescence assay was performed using a Dual-Luciferase Reporter Assay System (Promega) and Victor ×4 Multilabel Plate Reader (PerkinElmer, Waltham, MA) according to the manufacturer's instructions. Empty plasmid was used as the negative control, and the fluorescence intensity of firefly luciferase was normalized to the fluorescence intensity of Renilla luciferase. The values of the half-maximal inhibitory concentrations ($IC_{50}$) were calculated with the use of the GraphPad/SPSS software.

### RNA isolation and RT-qPCR

Total RNA was isolated using TRIzol Reagent (Thermo Fisher Scientific) and Phenol equilibrated, stabilized chloroform:isoamyl alcohol 25:24:1 (PanReac Applichem, Barcelona, Spain). A DeNovix Nanodrop Spectrophotometer was used to measure the RNA concentration. A total of 500 ng of total RNA was transcribed to cDNA using SuperScript III Reverse Transcriptase (Invitrogen) at 55°C. RT-qPCR was performed in a the CFX Connect Real-Time PCR Detection System (Bio-Rad, Hercules, CA) using SsoAdvanced Universal SYBR Green Supermix (Bio-Rad) with β-actin as the reference gene under the following thermal cycling conditions: denaturation at 95°C for 30 s followed by 40 cycles of denaturation at 95°C for 15 s and annealing at 60°C for 30 s. Sequences of specific primers are listed

**Figure 5. Lack of significant off-target effects after intrastriatal injection of amiR136-A2**

(A) Body weight was measured twice at 5-week intervals throughout the experimental period. No differences between the treated groups were observed in the amiRNA experiment. amiR136-A2 treatment decreased the weight of the spleen at both doses; p values: * <0.05; ** <0.01; **** <0.0001 (n = 8). For body weight analysis, two-way ANOVA was used, and for spleen weight analysis, one-way ANOVA was used; both were followed by Tukey's test. (B) IHC staining for Iba1 to show microglial activation, with GFAP to show astrocyte activity, with NeuN as a marker for neurons and with DARPP-32, which is specific for medium spiny neurons (MSNs). (C) RT-qPCR analysis of *Gfap* and *Iba1* transcript levels. (D) Western blot analysis of RBM33 and HCN1 proteins containing pure or interrupted CAG repeats in the corresponding genes. The signal intensities of the protein bands were normalized to those of calnexin (n = 8). (E) Analysis of the mRNA transcript level of the predicted off-target genes *Ppp1r3f, Maml1, Golga4, Ccdc177*, *Soga3*, and *Th*. The bars on the graph show the mean mRNA levels ± SEMs. Control – untreated YAC128 mice.

in Table S3. Gene expression levels were normalized to those in SCR-treated mice.

#### Bioinformatic analysis

To identify A2 off-target sequences, we mapped the A2 sequence to the mouse genome. We used bowtie (version 1.2.3) with the -a (all alignments) and -v 3 (max 3 mismatches) options. The MM10 genome assembly from University of California, Santa Cruz (UCSC) was used as the reference assembly. Python scripts were used to filter the results. By this analysis, we selected six genes (see Table S2) with full complementarity to A2.

#### Western blotting

Western blot analysis for HTT protein expression isolated from cell culture was performed as previously described.[24] Briefly, 30 μg of total protein was separated on a Tris-acetate SDS-polyacrylamide gel (1.5 cm, 4% stacking gel/4.5 cm, 5% resolving gel, acrylamide:bis-acrylamide ratio of 49:1) in XT Tricine buffer (Bio-Rad) at 135 V in an ice-water bath. For proteins isolated from mouse brains, NuPAGE Tris-Acetate 3%–8% Protein Gel (Thermo Fisher Scientific) in NuPAGE Tris-Acetate SDS Running Buffer (Thermo Fisher Scientific) were used. After electrophoresis, the proteins were transferred overnight to a nitrocellulose membrane (Sigma-Aldrich) by the wet transfer method. The primary and secondary antibodies were used in PBS/0.1% Tween 20 buffer containing 5% nonfat milk. Immunoreactions were detected using Western Bright Quantum HRP Substrate (Advansta, Menlo Park, CA). Protein bands were scanned directly from the membrane using a camera, and band densities were quantified using a Gel-Pro Analyzer (Media Cybernetics). Plectin or calnexin was used as the reference protein. A list of all antibodies used is provided in Table S4.

#### Small RNA next-generation sequencing and data analysis

Total RNA was isolated (TRI reagent) from HEK293T cells at 24 h post transfection, and the RNA quality was analyzed with an Agilent 2100 Bioanalyzer (RNA Nano Chip, Agilent, Santa Clara, CA). Small RNA sequencing was performed by CeGaT (Tubingen, Germany) using an Illumina HiSeq2500 with 1 × 50 base pair reads. Demultiplexing of the sequencing reads was performed with Illumina bcl2fastq (2.19) software. Adapter trimming was performed with Skewer (version 0.2.2).[52]

The reads in FASTQ format were then subjected to length filtering using a custom Python script, retaining only sequences longer than 15 nucleotides. Then, the reads were filtered for quality using the fastq_quality_filter tool in the FASTX-Toolkit package (http://hannonlab.cshl.edu/fastx_toolkit/). We applied the parameters −q20 and −p9, with which only reads having 95% of the bases with a Phred quality score ≥20 were retained. Through quality filtering, between 5% and 6% of the reads from each sample were discarded. Then, we removed redundant data with the fastx_collapse tool in the same package. The reads were finally mapped against the sequences of our shRNA constructs using bowtie, with no mismatches allowed. Finally, with an in-house Python script, the alignments were parsed and displayed in a graphical form for manual inspection.

#### Animal model and housing

All experiments were performed on YAC128 transgenic (FVB-Tg(YAC128)53Hay/J) and WT (FVB/NJ) mice maintained on the FVB/NJ strain background.[53] Mice were acquired from The Jackson Laboratory and bred in the animal facility of Center for Advanced Technologies Adam Mickiewicz University in Poznan (CAT AMU) where the experiments were conducted. All experiments were approved by the Local Ethical Committee for Animal Experiments (approval no. 45/2018 given on 22.11.2018). Animals were housed under specific pathogen-free conditions, and their health was monitored on a 3-month basis. Mice were housed in individually ventilated cages with access to water and food *ad libitum*.

#### Intrastriatal delivery of AAVs

In the treated groups, we stereotaxically injected 3 μL of AAV5 vectors into the striatum of both hemispheres at specific coordinates (AP + 0.7 mm, ML ± 1.7 mm, and DV −3.5 mm from the bregma) using a Hamilton gauge syringe over a 10-min period (0.3 μL/min). In the pilot experiment (n = 10 per construct), mice were injected with 3 μL of AAV5 vectors unilaterally into the right hemisphere. All surgeries were performed under inhaled isoflurane anesthesia, and mice were placed on a heating pad to prevent hypothermia. The wound was covered with antibiotics to prevent infection. After surgery, mice were injected subcutaneously with a nonsteroidal anti-inflammatory drug (meloxicam) and transferred to preheated cages for recovery. The health of mice was monitored for at least 2 h postsurgery and afterward on a daily basis. WT littermate mice were used as healthy controls in the shA2 experiment.

#### Animal perfusion and tissue collection

Mice were subjected to cardiac perfusion with PBS to remove all blood and were then perfused with 4% paraformaldehyde solution. After 24 h, brains were transferred to 30% sucrose for 72 h. Then, tissues were sectioned into 25-μm sections using a cryostat at −16°C and mounted on SuperFrost Plus slides (Thermo Scientific).

#### Immunohistochemistry

Heat-induced antigen retrieval was performed. Sections were incubated in citrate buffer (pH 6.0) for 30 min in a boiling water bath and were then placed in ice-cold TBS-T. Then, sections were blocked with 4% normal goat serum in TBS-T for 1 h. For immunofluorescence staining, sections were incubated overnight at 4°C with the primary antibodies (listed in Table S4) and subsequently with the corresponding secondary antibodies. Sections were mounted using ProLong Gold Antifade mounting reagent with DAPI (Thermo Fisher P36941).

For aggregate staining, EM48 primary antibody (Sigma-Aldrich) and an ImmPRESS Horse Anti-Mouse IgG PLUS Polymer Kit (Vector Laboratories, Burlingame, CA) were used according to the manufacturer's instructions. Images were acquired with a Leica SP5 confocal microscope. ImageJ Software (NIH, Bethesda, MD) was used for aggregate quantification. The counts were made from eight images from each hemisphere (sections separated by 50 μm).

### Behavioral tests

We performed motor function tests (rotarod, static rod, cylinder, grip strength tests) for 20 weeks post injection to evaluate the effect of presymptomatic treatment on the HD phenotype.

#### *Rotarod test*

We used an accelerating rotarod protocol (Ugo-Basile) to test motor coordination and learning capabilities. The acceleration ranged from 3 to 40 rpm over 5 min. After the training period (three trials per day for 3 days), mice were tested with three consecutive trials in a single day. The rotarod was wiped clean with ethanol between each subject and trial.

#### *Static rod test*

To further assess motor deficits in treated YAC128 animals, a static rod test was employed. Mice were placed on four different rods with a specific diameter (28 mm, 21 mm, 17 mm, and 10 mm) and a length of 60 cm facing outward and 100 cm above the bottom surface. Fall protection was provided by a soft cushion below the rod. The time to turn to safety and time to traverse the rod were recorded. The test was repeated two times on consecutive days.

#### *Cylinder (beaker) test*

Mice were placed in a transparent beaker with a 90-mm diameter and a height of 125 mm for 3 min. During that time, rearings were counted. For rearing, an animal must be standing on two paws and standing straight with at least one paw touching the wall of the glass cylinder.

### Statistical analysis (behavioral)

Statistical analysis of the obtained data was performed with GraphPad/SPSS software. Based on experience and the literature, the majority of experiments would have a power of 80% to achieve a significance level of 0.05. Data are presented as the SEM. Tests to check for a normal distribution were performed. If a normal distribution was confirmed, the data were analyzed by ANOVA or a t test; if the normality assumption was violated, the data were analyzed using Kruskal-Wallis and Mann-Whitney tests, with $p < 0.05$ considered significant. For behavioral testing, when time dependency was considered, two-way ANOVA was performed with additional correction for multiple comparisons with the Holm-Sidak test.

### Statistical analysis (molecular)

All experiments were repeated at least three times. The statistical significance of silencing was assessed using a one-sample t test, with an arbitrary value of 1 assigned to the cells treated with control. Selected data were compared using an unpaired t test. Two-tailed p values of <0.05 were considered significant. Signal intensities of the protein bands were normalized to those of calnexin and compared using Student's t test. The bars on the graphs indicate the mean protein levels ±SEM. p values are indicated by asterisks (*$p < 0.03$, **$p < 0.002$, ***$p < 0.0002$, ****$p < 0.0001$).

## SUPPLEMENTAL INFORMATION

Supplemental information can be found online at https://doi.org/10.1016/j.omtn.2022.04.031.

## ACKNOWLEDGMENTS

This study was supported by research grants from the National Science Center PL [2015/18/E/NZ2/00678; 2016/21/D/NZ4/00478; 2019/35/O/NZ1/03535] and Dystrogen Gene Therapies Inc, Chicago, IL. We thank Magdalena Otrocka, Gabriela Kramer-Marek, and Adam Plewinski for technical support. The microscopy analysis was performed in the Laboratory of Subcellular Structures Analysis at the Institute of Bioorganic Chemistry, PAS, in Poznan.

## AUTHOR CONTRIBUTIONS

M.O., M.F., L.P., J.S.Z., and A.K.Z. designed the research; A.K.Z., L.P., M.P., J.S.Z., and D.W. performed the experiments; A.K.Z., L.P., M.P., and M.O. analyzed the data and interpreted the results of the experiments; A.K.Z. and L.P. prepared the figures; M.O., A.K.Z., and L.P. drafted, edited, and revised the manuscript; A.K.Z., L.P., M.P., J.S.Z., D.W., M.F., and M.O. approved the final version of manuscript.

## DECLARATION OF INTERESTS

Marta Olejniczak (MO) is a coinventor on patents (US9970004B2 and US10329566B2) for the use of the RNAi approach in the treatment of diseases induced by expansion of trinucleotide CAG repeats.

## REFERENCES

1. MacDonald, M.E., Ambrose, C.M., Duyao, M.P., Myers, R.H., Lin, C., Srinidhi, L., Barnes, G., Taylor, S.A., James, M., Groot, N., et al. (1993). A novel gene containing a trinucleotide repeat that is expanded and unstable on Huntington's disease chromosomes. Cell *72*, 971–983. https://doi.org/10.1016/0092-8674(93)90585-e.
2. Ross, C.A. (2002). Polyglutamine pathogenesis: emergence of unifying mechanisms for Huntington's disease and related disorders. Neuron *35*, 819–822. https://doi.org/10.1016/s0896-6273(02)00872-3.
3. Duyao, M., Ambrose, C., Myers, R., Novelletto, A., Persichetti, F., Frontali, M., Folstein, S., Ross, C., Franz, M., and Abbott, M. (1993). Trinucleotide repeat length instability and age of onset in Huntington's disease. Nat. Genet. *4*, 387–392. https://doi.org/10.1038/ng0893-387.
4. Monckton, D.G. (2021). The contribution of somatic expansion of the CAG repeat to symptomatic development in Huntington's disease: a historical perspective. J. Huntingtons Dis. *10*, 7–33. https://doi.org/10.3233/JHD-200429.
5. Zeitlin, S., Liu, J.P., Chapman, D.L., Papaioannou, V.E., and Efstratiadis, A. (1995). Increased apoptosis and early embryonic lethality in mice nullizygous for the Huntington's disease gene homologue. Nat. Genet. *11*, 155–163. https://doi.org/10.1038/ng1095-155.
6. Nasir, J., Floresco, S.B., O'Kusky, J.R., Diewert, V.M., Richman, J.M., Zeisler, J., Borowski, A., Marth, J.D., Phillips, A.G., and Hayden, M.R. (1995). Targeted disruption of the Huntington's disease gene results in embryonic lethality and behavioral and morphological changes in heterozygotes. Cell *81*, 811–823. https://doi.org/10.1016/0092-8674(95)90542-1.
7. Van Raamsdonk, J.M., Murphy, Z., Slow, E.J., Leavitt, B.R., and Hayden, M.R. (2005). Selective degeneration and nuclear localization of mutant huntingtin in the YAC128 mouse model of Huntington disease. Hum. Mol. Genet. *14*, 3823–3835. https://doi.org/10.1093/hmg/ddi407.
8. Novak, M.J.U., and Tabrizi, S.J. (2010). Huntington's disease. BMJ *340*, c3109. https://doi.org/10.1136/bmj.c3109.
9. Gutekunst, C.-A., Li, S.-H., Yi, H., Mulroy, J.S., Kuemmerle, S., Jones, R., Rye, D., Ferrante, R.J., Hersch, S.M., and Li, X.-J. (1999). Nuclear and neuropil aggregates in Huntington's disease: relationship to neuropathology. J. Neurosci. *19*, 2522–2534. https://doi.org/10.1523/jneurosci.19-07-02522.1999.
10. Sathasivam, K., Neueder, A., Gipson, T.A., Landles, C., Benjamin, A.C., Bondulich, M.K., Smith, D.L., Faull, R.L.M., Roos, R.A.C., Howland, D., et al. (2013). Aberrant

splicing of HTT generates the pathogenic exon 1 protein in Huntington disease. PNAS *110*, 2366–2370. https://doi.org/10.1073/pnas.1221891110.

11. Neueder, A., Landles, C., Ghosh, R., Howland, D., Myers, R.H., Faull, R.L.M., Tabrizi, S.J., and Bates, G.P. (2017). The pathogenic exon 1 HTT protein is produced by incomplete splicing in Huntington's disease patients. Sci. Rep. *7*, 1307. https://doi.org/10.1038/s41598-017-01510-z.
12. Pan, L., and Feigin, A. (2021). Huntington's disease: new frontiers in therapeutics. Curr. Neurol. Neurosci. Rep. *21*, 10. https://doi.org/10.1007/s11910-021-01093-3.
13. Dietrich, P., Johnson, I.M., Alli, S., and Dragatsis, I. (2017). Elimination of huntingtin in the adult mouse leads to progressive behavioral deficits, bilateral thalamic calcification, and altered brain iron homeostasis. PLoS Genet. *13*, e1006846. https://doi.org/10.1371/journal.pgen.1006846.
14. Tabrizi, S.J., Leavitt, B.R., Landwehrmeyer, G.B., Wild, E.J., Saft, C., Barker, R.A., Blair, N.F., Craufurd, D., Priller, J., Rickards, H., et al. (2019). Targeting huntingtin expression in patients with Huntington's disease. N. Engl. J. Med. *380*, 2307–2316. https://doi.org/10.1056/nejmoa1900907.
15. Alterman, J.F., Godinho, B.M.D.C., Hassler, M.R., Ferguson, C.M., Echeverria, D., Sapp, E., Haraszti, R.A., Coles, A.H., Conroy, F., Miller, R., et al. (2019). A divalent siRNA chemical scaffold for potent and sustained modulation of gene expression throughout the central nervous system. Nat. Biotechnol. *37*, 884–894. https://doi.org/10.1038/s41587-019-0205-0.
16. Kotowska-Zimmer, A., Pewinska, M., and Olejniczak, M. (2021). Artificial miRNAs as therapeutic tools: challenges and opportunities. Wiley Interdiscip. Rev. RNA *12*, e1640.
17. Evers, M.M., Miniarikova, J., Juhas, S., Vallès, A., Bohuslavova, B., Juhasova, J., Skalnikova, H.K., Vodicka, P., Valekova, I., Brouwers, C., et al. (2018). AAV5-miHTT gene therapy demonstrates broad distribution and strong human mutant huntingtin lowering in a Huntington's disease minipig model. Mol. Ther. *26*, 2163–2177. https://doi.org/10.1016/j.ymthe.2018.06.021.
18. Miniarikova, J., Zimmer, V., Martier, R., Brouwers, C.C., Pythoud, C., Richetin, K., Rey, M., Lubelski, J., Evers, M.M., van Deventer, S.J., et al. (2017). AAV5-miHTT gene therapy demonstrates suppression of mutant huntingtin aggregation and neuronal dysfunction in a rat model of Huntington's disease. Gene Ther. *24*, 630–639. https://doi.org/10.1038/gt.2017.71.
19. Caron, N.S., Southwell, A.L., Brouwers, C.C., Cengio, L.D., Xie, Y., Black, H.F., Anderson, L.M., Ko, S., Zhu, X., van Deventer, S.J., et al. (2020). Potent and sustained huntingtin lowering via AAV5 encoding miRNA preserves striatal volume and cognitive function in a humanized mouse model of Huntington disease. Nucleic Acids Res. *48*, 36–54. https://doi.org/10.1093/nar/gkz976.
20. Pfister, E.L., Chase, K.O., Sun, H., Kennington, L.A., Conroy, F., Johnson, E., Miller, R., Borel, F., Aronin, N., and Mueller, C. (2017). Safe and efficient silencing with a Pol II, but not a Pol lII, promoter expressing an artificial miRNA targeting human huntingtin. Mol. Ther. Nucleic Acids *7*, 324–334. https://doi.org/10.1016/j.omtn.2017.04.011.
21. Stanek, L.M., Sardi, S.P., Mastis, B., Richards, A.R., Treleaven, C.M., Taksir, T., Misra, K., Cheng, S.H., and Shihabuddin, L.S. (2014). Silencing mutant huntingtin by adeno-associated virus-mediated RNA interference ameliorates disease manifestations in the YAC128 mouse model of Huntington's disease. Hum. Gene Ther. *25*, 461–474. https://doi.org/10.1089/hum.2013.200.
22. McBride, J.L., Pitzer, M.R., Boudreau, R.L., Dufour, B., Hobbs, T., Ojeda, S.R., and Davidson, B.L. (2011). Preclinical safety of RNAi-mediated HTT suppression in the rhesus macaque as a potential therapy for Huntington's disease. Mol. Ther. *19*, 2152–2162. https://doi.org/10.1038/mt.2011.219.
23. Fiszer, A., Olejniczak, M., Galka-Marciniak, P., Mykowska, A., and Krzyzosiak, W.J. (2013). Self-duplexing CUG repeats selectively inhibit mutant huntingtin expression. Nucleic Acids Res. *41*, 10426–10437. https://doi.org/10.1093/nar/gkt825.
24. Kotowska-Zimmer, A., Ostrovska, Y., and Olejniczak, M. (2020). Universal RNAi triggers for the specific inhibition of mutant huntingtin, atrophin-1, ataxin-3, and ataxin-7 expression. Mol. Ther. Nucleic Acids *19*, 562–571. https://doi.org/10.1016/j.omtn.2019.12.012.
25. Ciesiolka, A., Stroynowska-Czerwinska, A., Joachimiak, P., Ciolak, A., Kozlowska, E., Michalak, M., Dabrowska, M., Olejniczak, M., Raczynska, K.D., Zielinska, D., et al. (2021). Artificial miRNAs targeting CAG repeat expansion in ORFs cause rapid deadenylation and translation inhibition of mutant transcripts. Cell Mol. Life Sci. *78*, 1577–1596. https://doi.org/10.1007/s00018-020-03596-7.
26. Hu, J., Liu, J., and Corey, D.R. (2010). Allele-selective inhibition of huntingtin expression by switching to an miRNA-like RNAi mechanism. Chem. Biol. *17*, 1183–1188. https://doi.org/10.1016/j.chembiol.2010.10.013.
27. Yoda, M., Cifuentes, D., Izumi, N., Sakaguchi, Y., Suzuki, T., Giraldez, A.J., and Tomari, Y. (2013). Poly(A)-Specific ribonuclease mediates 3′-end trimming of argonaute2-cleaved precursor MicroRNAs. Cell Rep. *5*, 715–726. https://doi.org/10.1016/j.celrep.2013.09.029.
28. Galka-Marciniak, P., Olejniczak, M., Starega-Roslan, J., Szczesniak, M.W., Makalowska, I., and Krzyzosiak, W.J. (2016). siRNA release from pri-miRNA scaffolds is controlled by the sequence and structure of RNA. Biochim. Biophys. Acta *1859*, 639–649. https://doi.org/10.1016/j.bbagrm.2016.02.014.
29. Jopling, C. (2012). Liver-specific microRNA-122: biogenesis and function. RNA Biol. *9*, 137–142. https://doi.org/10.4161/rna.18827.
30. Kozomara, A., Birgaoanu, M., and Griffiths-Jones, S. (2019). miRBase: from microRNA sequences to function. Nucleic Acids Res. *47*, D155–D162. https://doi.org/10.1093/nar/gky1141.
31. Cheloufi, S., Dos Santos, C.O., Chong, M.M.W., and Hannon, G.J. (2010). A Dicer-independent miRNA biogenesis pathway that requires Ago catalysis. Nature *465*, 584–589. https://doi.org/10.1038/nature09092.
32. Pouladi, M.A., Stanek, L.M., Xie, Y., Franciosi, S., Southwell, A.L., Deng, Y., Butland, S., Zhang, W., Cheng, S.H., Shihabuddin, L.S., and Hayden, M.R. (2012). Marked differences in neurochemistry and aggregates despite similar behavioural and neuropathological features of Huntington disease in the full-length BACHD and YAC128 mice. Hum. Mol. Genet. *21*, 2219–2232. https://doi.org/10.1093/hmg/dds037.
33. Slow, E.J., van Raamsdonk, J., Rogers, D., Coleman, S.H., Graham, R.K., Deng, Y., Oh, R., Bissada, N., Hossain, S.M., Yang, Y.-Z., et al. (2003). Selective striatal neuronal loss in a YAC128 mouse model of Huntington disease. Hum. Mol. Genet. *12*, 1555–1567. https://doi.org/10.1093/hmg/ddg169.
34. McBride, J.L., Boudreau, R.L., Harper, S.Q., Staber, P.D., Monteys, A.M., Martins, I., Gilmore, B.L., Burstein, H., Peluso, R.W., Polisky, B., et al. (2008). Artificial miRNAs mitigate shRNA-mediated toxicity in the brain: implications for the therapeutic development of RNAi. Proc. Natl. Acad. Sci. U S A *105*, 5868–5873. https://doi.org/10.1073/pnas.0801775105.
35. Grimm, D. (2011). The dose can make the poison: lessons learned from adverse in vivo toxicities caused by RNAi overexpression. Silence *2*, 8. https://doi.org/10.1186/1758-907x-2-8.
36. Martin, J.N., Wolken, N., Brown, T., Dauer, W.T., Ehrlich, M.E., and Gonzalez-Alegre, P. (2011). Lethal toxicity caused by expression of shRNA in the mouse striatum: implications for therapeutic design. Gene Ther. *18*, 666–673. https://doi.org/10.1038/gt.2011.10.
37. Van Raamsdonk, J.M., Gibson, W.T., Pearson, J., Murphy, Z., Lu, G., Leavitt, B.R., and Hayden, M.R. (2006). Body weight is modulated by levels of full-length Huntingtin. Hum. Mol. Genet. *15*, 1513–1523. https://doi.org/10.1093/hmg/ddl072.
38. Van Raamsdonk, J.M., Pearson, J., Slow, E.J., Hossain, S.M., Leavitt, B.R., and Hayden, M.R. (2005). Cognitive dysfunction precedes neuropathology and motor abnormalities in the YAC128 mouse model of Huntington's disease. J. Neurosci. *25*, 4169–4180. https://doi.org/10.1523/jneurosci.0590-05.2005.
39. Van Raamsdonk, J.M., Murphy, Z., Selva, D.M., Hamidizadeh, R., Pearson, J., Petersén, Á., Björkqvist, M., Muir, C., Mackenzie, I.R., Hammond, G.L., et al. (2007). Testicular degeneration in Huntington disease. Neurobiol. Dis. *26*, 512–520. https://doi.org/10.1016/j.nbd.2007.01.006.
40. Southwell, A.L., Ko, J., and Patterson, P.H. (2009). Intrabody gene therapy ameliorates motor, cognitive, and neuropathological symptoms in multiple mouse models of Huntington's disease. J. Neurosci. *29*, 13589–13602. https://doi.org/10.1523/jneurosci.4286-09.2009.
41. Pouladi, M.A., Graham, R.K., Karasinska, J.M., Xie, Y., Santos, R.D., Petersén, A., and Hayden, M.R. (2009). Prevention of depressive behaviour in the YAC128 mouse model of Huntington disease by mutation at residue 586 of huntingtin. Brain *132*, 919–932. https://doi.org/10.1093/brain/awp006.
42. Zeitler, B., Froelich, S., Marlen, K., Shivak, D.A., Yu, Q., Li, D., Pearl, J.R., Miller, J.C., Zhang, L., Paschon, D.E., et al. (2019). Allele-selective transcriptional repression of

mutant HTT for the treatment of Huntington's disease. Nat. Med. *25*, 1131–1142. https://doi.org/10.1038/s41591-019-0478-3.

43. Monteys, A.M., Wilson, M.J., Boudreau, R.L., Spengler, R.M., and Davidson, B.L. (2015). Artificial miRNAs targeting mutant huntingtin show preferential silencing in vitro and in vivo. Mol. Ther. Nucleic Acids *4*, e234. https://doi.org/10.1038/mtna.2015.7.

44. Miniarikova, J., Zanella, I., Huseinovic, A., van der Zon, T., Hanemaaijer, E., Martier, R., Koornneef, A., Southwell, A.L., Hayden, M.R., van Deventer, S.J., et al. (2016). Design, characterization, and lead selection of therapeutic miRNAs targeting huntingtin for development of gene therapy for Huntington's disease. Mol. Ther. Nucleic Acids *5*, e297. https://doi.org/10.1038/mtna.2016.7.

45. Grimm, D., Streetz, K.L., Jopling, C.L., Storm, T.A., Pandey, K., Davis, C.R., Marion, P., Salazar, F., and Kay, M.A. (2006). Fatality in mice due to oversaturation of cellular microRNA/short hairpin RNA pathways. Nature *441*, 537–541. https://doi.org/10.1038/nature04791.

46. Krauß, S., Griesche, N., Jastrzebska, E., Chen, C., Rutschow, D., Achmüller, C., Dorn, S., Boesch, S.M., Lalowski, M., Wanker, E., et al. (2013). Translation of HTT mRNA with expanded CAG repeats is regulated by the MID1-PP2A protein complex. Nat. Commun. *4*, 1511. https://doi.org/10.1038/ncomms2514.

47. Wojciechowska, M., Olejniczak, M., Galka-Marciniak, P., Jazurek, M., and Krzyzosiak, W.J. (2014). RAN translation and frameshifting as translational challenges at simple repeats of human neurodegenerative disorders. Nucleic Acids Res. *42*, 11849–11864. https://doi.org/10.1093/nar/gku794.

48. Ghosh, R., Wood-Kaczmar, A., Dobson, L., Smith, E.J., Sirinathsinghji, E.C., Kriston-Vizi, J., Hargreaves, I.P., Heaton, R., Herrmann, F., Abramov, A.Y., et al. (2020). Expression of mutant exon 1 huntingtin fragments in human neural stem cells and neurons causes inclusion formation and mitochondrial dysfunction. FASEB J. *34*, 8139–8154. https://doi.org/10.1096/fj.201902277rr.

49. Burger, C., Gorbatyuk, O.S., Velardo, M.J., Peden, C.S., Williams, P., Zolotukhin, S., Reier, P.J., Mandel, R.J., and Muzyczka, N. (2004). Recombinant AAV viral vectors pseudotyped with viral capsids from serotypes 1, 2, and 5 display differential efficiency and cell tropism after delivery to different regions of the central nervous system. Mol. Ther. *10*, 302–317. https://doi.org/10.1016/j.ymthe.2004.05.024.

50. Pietersz, K.L., Martier, R.M., Baatje, M.S., Liefhebber, J.M., Brouwers, C.C., Pouw, S.M., Fokkert, L., Lubelski, J., Petry, H., Martens, G.J.M., et al. (2021). Transduction patterns in the CNS following various routes of AAV-5-mediated gene delivery. Gene Ther. *28*, 435–446. https://doi.org/10.1038/s41434-020-0178-0.

51. Sogorb-Gonzalez, M., Vendrell-Tornero, C., Snapper, J., Stam, A., Keskin, S., Miniarikova, J., Spronck, E.A., de Haan, M., Nieuwland, R., Konstantinova, P., et al. (2021). Secreted therapeutics: monitoring durability of microRNA-based gene therapies in the central nervous system. Brain Commun. *3*. https://doi.org/10.1093/braincomms/fcab054.

52. Jiang, H., Lei, R., Ding, S.-W., and Zhu, S. (2014). Skewer: a fast and accurate adapter trimmer for next-generation sequencing paired-end reads. BMC Bioinf. *15*, 182. https://doi.org/10.1186/1471-2105-15-182.

53. Hodgson, J.G., Agopyan, N., Gutekunst, C.A., Leavitt, B.R., LePiane, F., Singaraja, R., Smith, D.J., Bissada, N., McCutcheon, K., Nasir, J., et al. (1999). A YAC mouse model for Huntington's disease with full-length mutant huntingtin, cytoplasmic toxicity, and selective striatal neurodegeneration. Neuron *23*, 181–192. https://doi.org/10.1016/s0896-6273(00)80764-3.

OMTN, Volume *28*

## Supplemental information

# A CAG repeat-targeting artificial miRNA lowers the mutant huntingtin level in the YAC128 model of Huntington's disease

**Anna Kotowska-Zimmer, Lukasz Przybyl, Marianna Pewinska, Joanna Suszynska-Zajczyk, Dorota Wronka, Maciej Figiel, and Marta Olejniczak**

# SUPPLEMENTAL INFORMATION

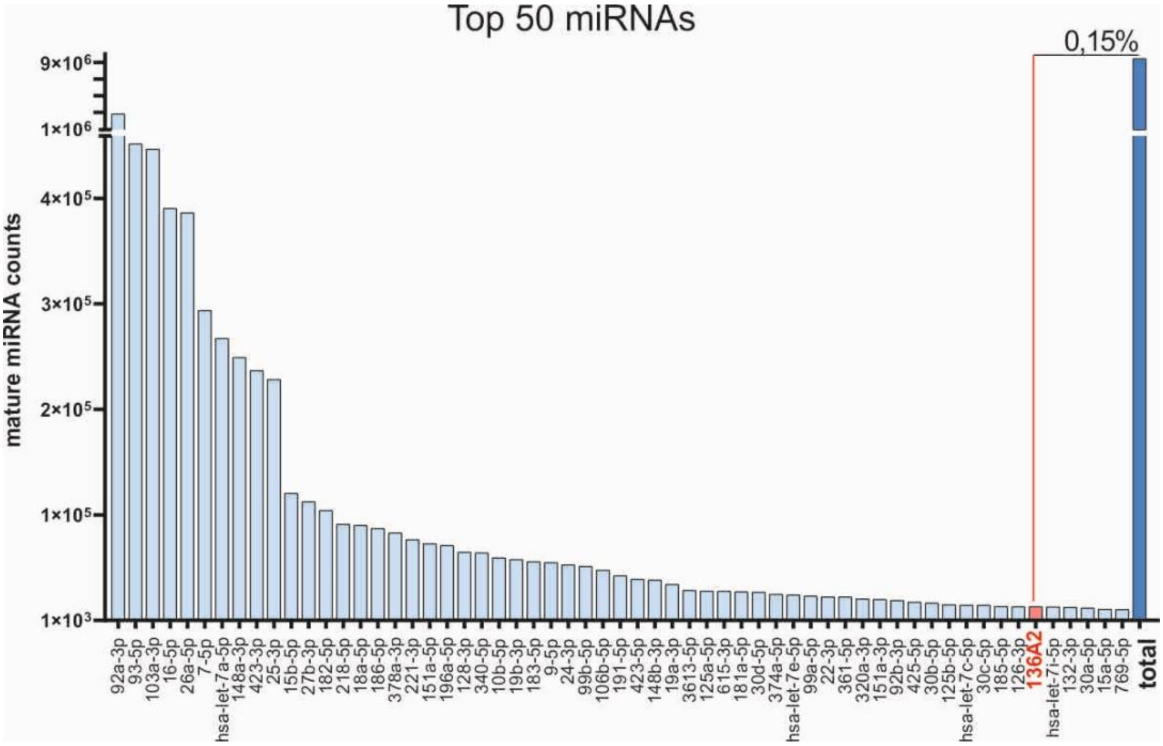


**Supplemental Figure S1.** Small RNA sequencing analysis of amiR136-A2-treated HEK293T cells. Top 50 most abundant miRNAs. The red bar indicates the mature amiR136-A2 count, and the last bar indicates the total mature miRNA count.

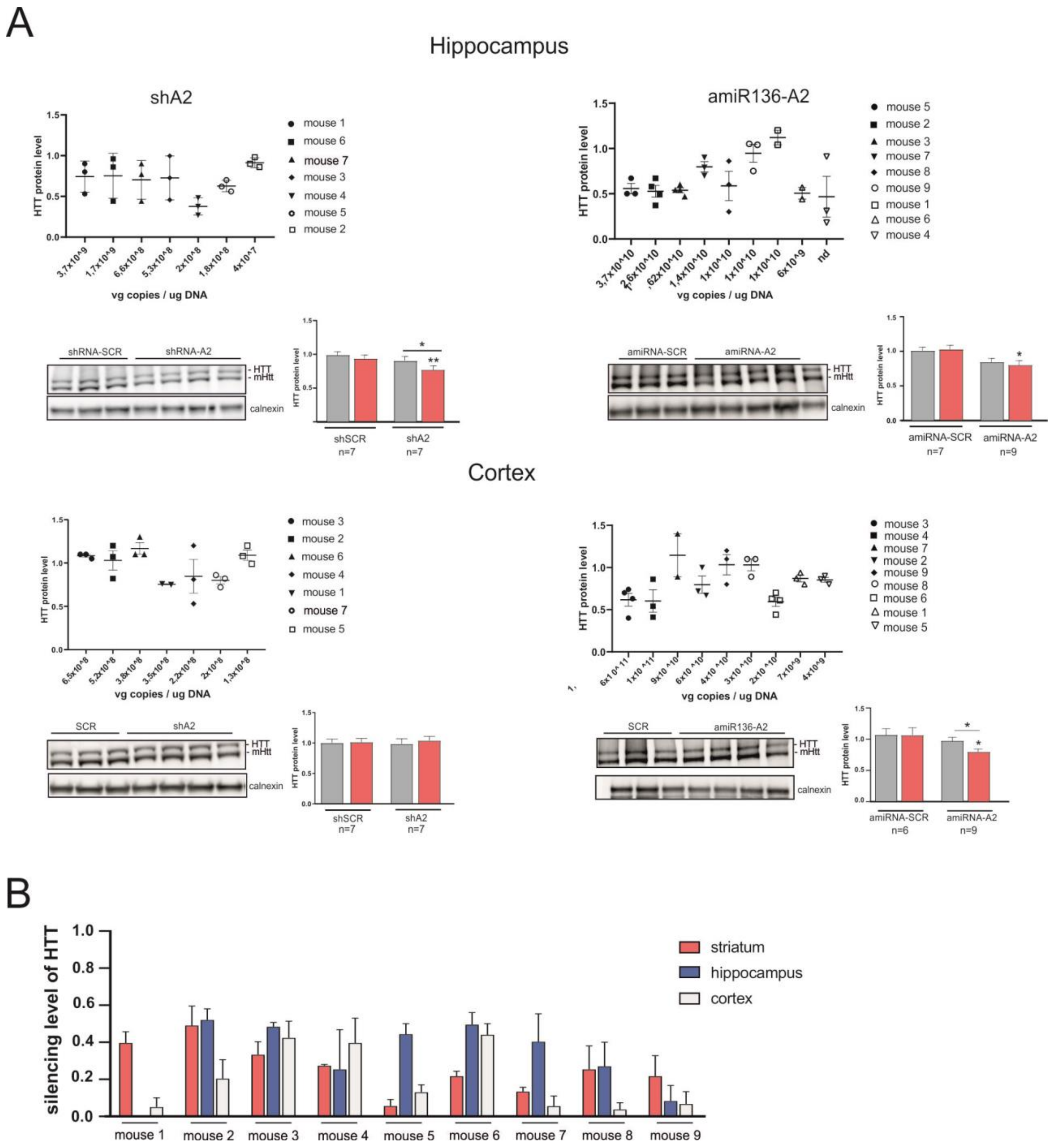


**Supplemental Figure S2.** Analysis of HTT protein silencing and AAV5 vector genome copies in the hippocampus and cortex of YAC128 mice. (A) qPCR to determine AAV5 genome copies (gc) in the brain structures of shA2- and amiR136-A2-injected mice (n=7 and n=9, respectively), one month post injection. Primers specific for the H1 and CAG promoters were used, and the gc values were calculated based on the standard curve. Western blot analysis of the HTT protein level. (B) Comparison of silencing efficiency in the striatum, hippocampus and cortex in amiR136-A2-treated mice. Signal intensities of the protein bands were normalized to those of calnexin and compared using Student's t-test. The bars on the graph indicate the mean protein levels ± SEMs. P values are indicated by asterisks (*p < 0.03, **p < 0.002).

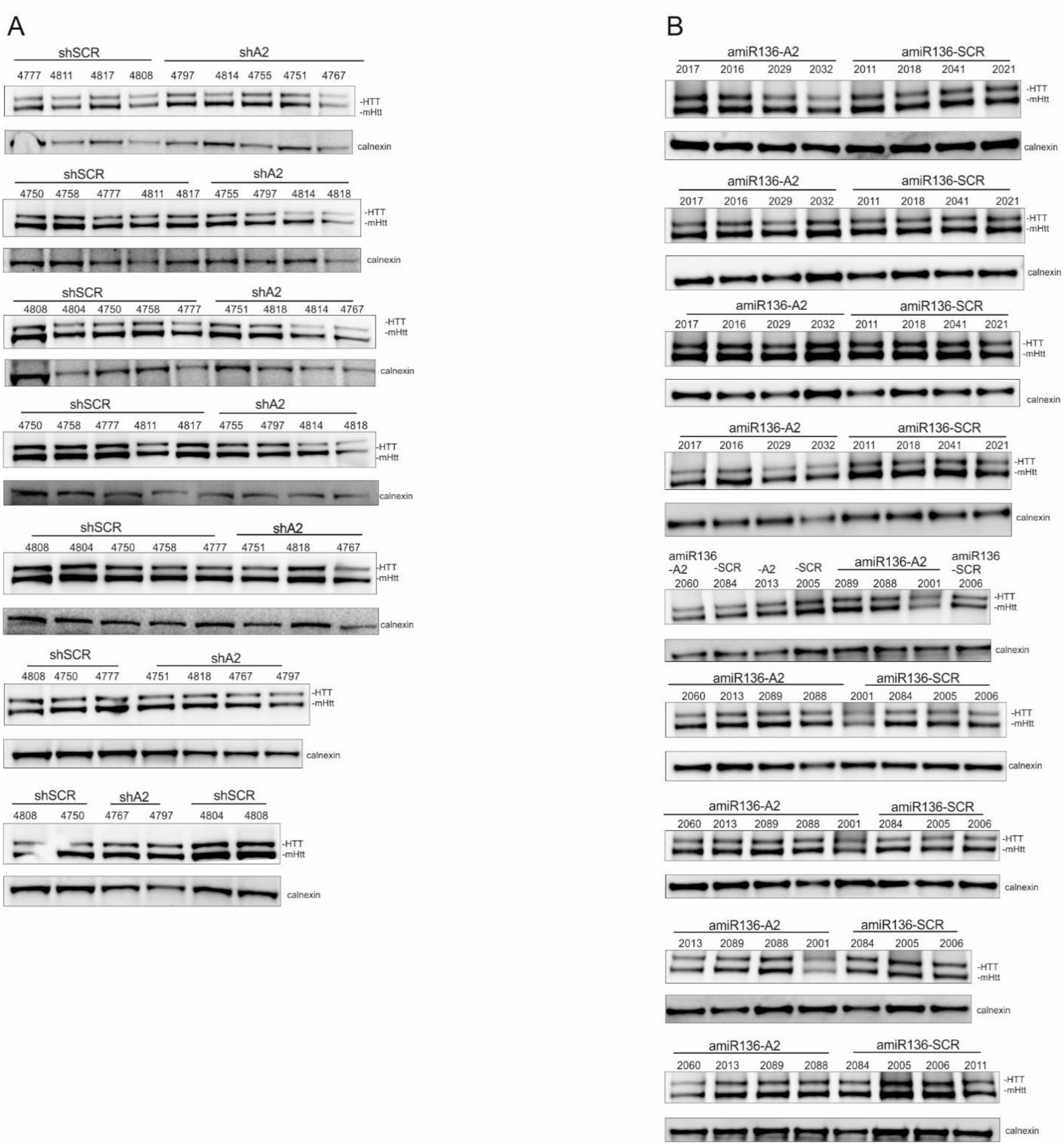


**Supplemental Figure S3.** Western blots used for quantification of HTT suppression in the striatum one month post injection of (A)AAV5-shA2 and (B) amiR136-A2.

A

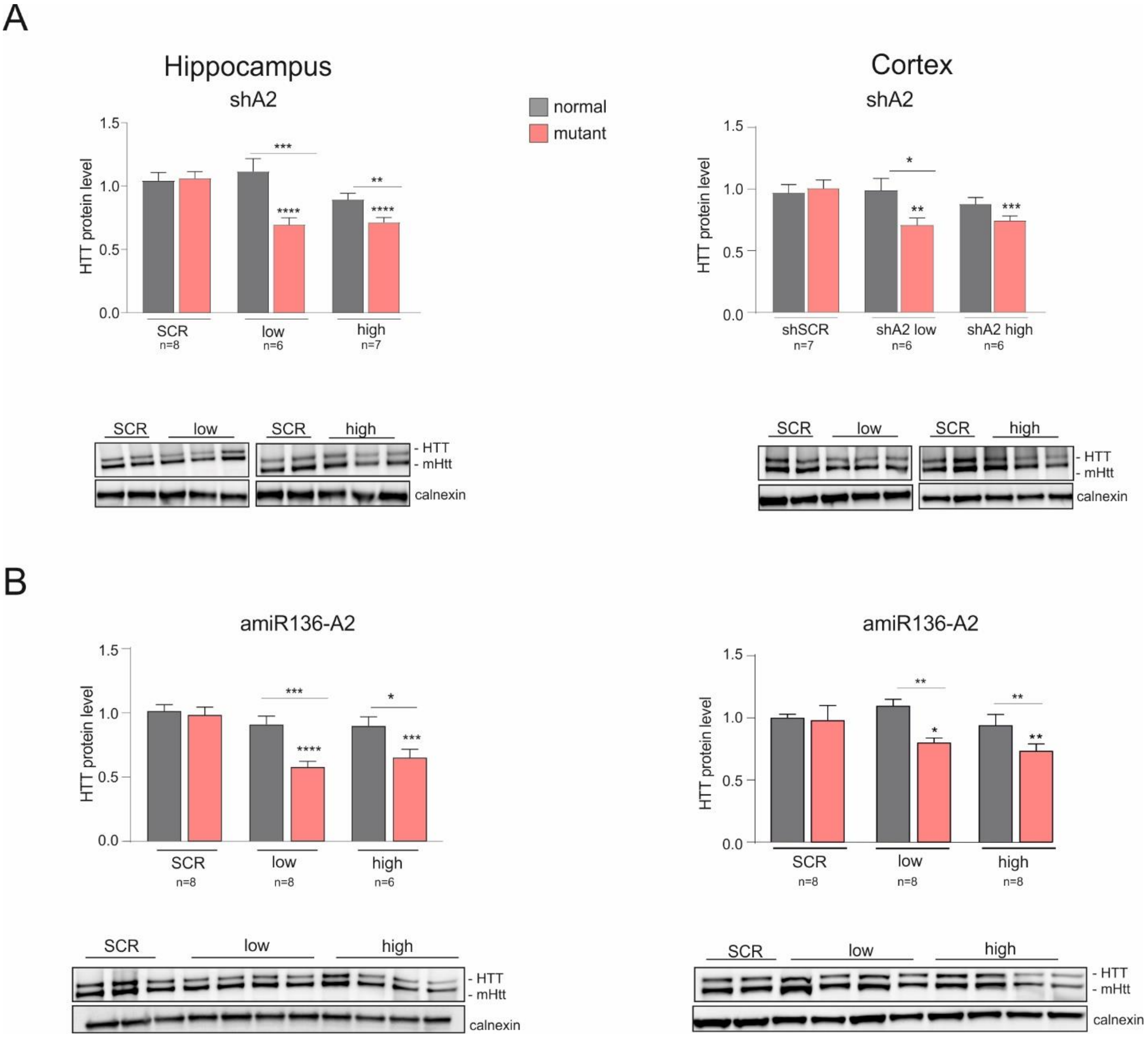


**Supplemental Figure S4.** Long-term analysis of the efficacy and allele selectivity of shA2 and amiR136-A2 in the hippocampus and cortex in treated mice. (**A**) Western blot analysis of the HTT protein level in the hippocampus and cortex 20 weeks post injection with AAV5-shA2. The shSCR construct was used as the reference control. (**B**) Western blot analysis of the HTT protein level in the hippocampus and cortex 20 weeks post injection with AAV5-amiR136-A2. The amiR136-SCR construct was used as the reference control. The bars on the graph indicate the mean protein levels ± SEMs. P values are indicated by asterisks (*p < 0.03, **p < 0.002, ***p < 0.0002, ****p<0.0001).

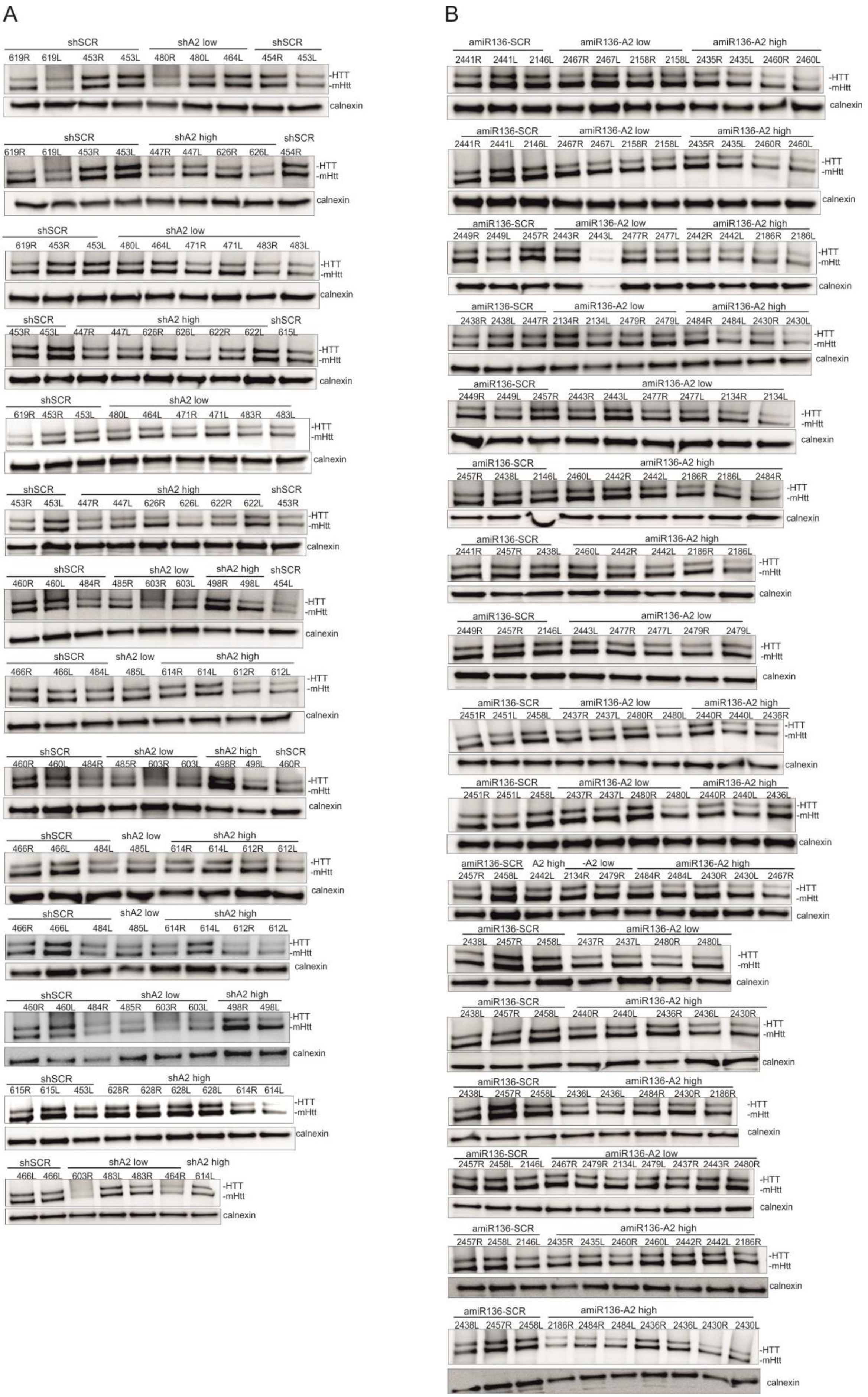
A
shSCR
shA2 low
shA2 high
-HTT
-mHtt
calnexin
B
amiR136-SCR
amiR136-A2 low
amiR136-A2 high
-HTT
-mHtt
calnexin

**Supplemental Figure S5.** Western blots used for quantification of HTT suppression in the striatum 20 weeks post injection with (**A**) AAV5-shA2 and (**B**) amiR136-A2. L- left hemisphere, R – right hemisphere

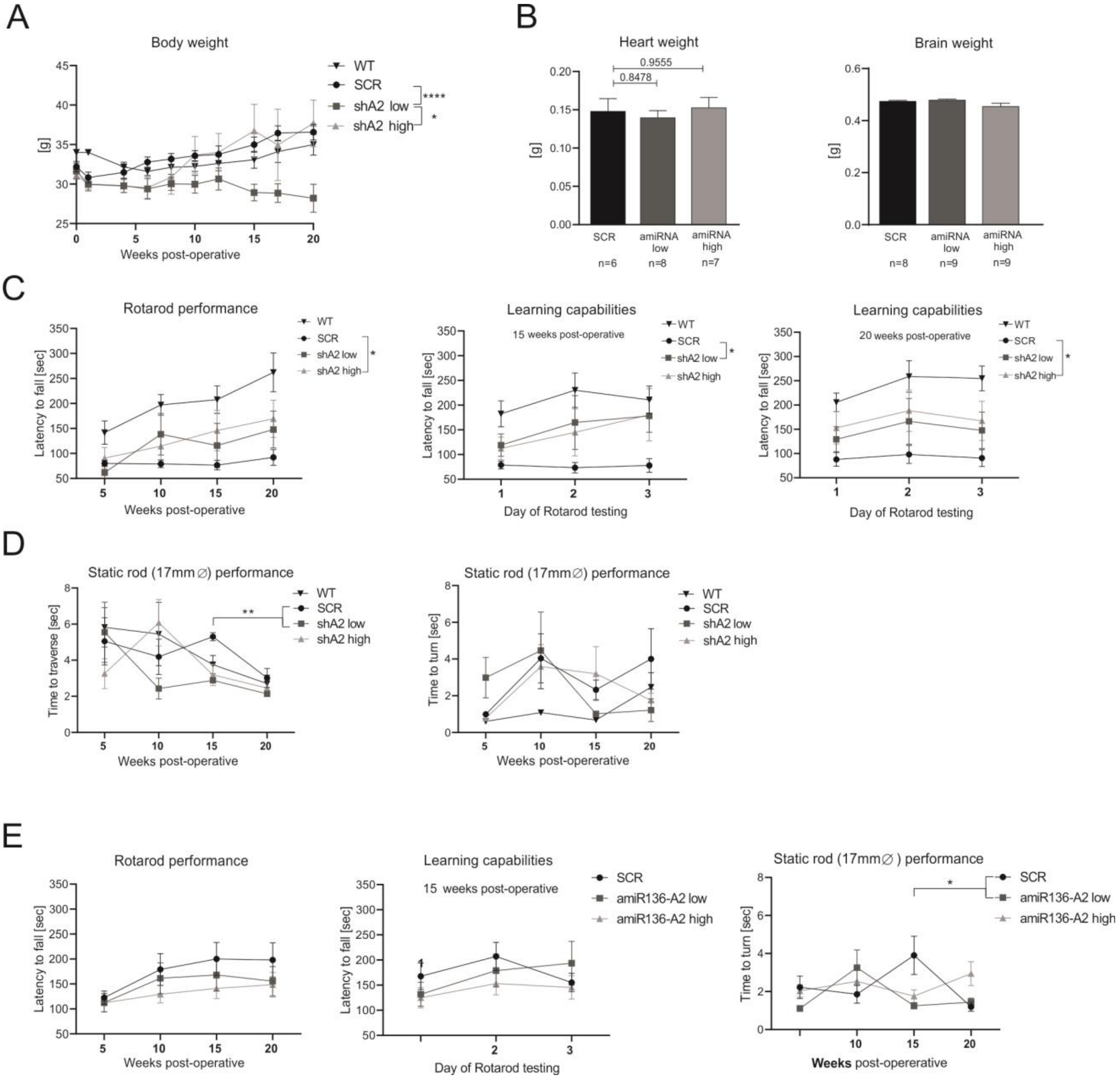


**Supplemental Figure S6.** The effects of shA2 and amiR136-A2 administration on body and organ weight and on behavior in YAC128 mice. (**A**) Body weight was measured twice at 5 week intervals throughout the experimental period and showed significant differences between the SCR- and shRNA-treated groups at both the low and high doses. WT animals were used as healthy controls. (**B**) The heart weight and brain weight did not change after amiR136-A2 treatment (**C**) When tested for 3 consecutive days, mice showed improvement in learning on the rotarod test toward the performance of healthy animals 15 weeks after injection with the low dose of shRNA (**D**) The results of the static rod test. Mice treated with shA2 traversed the 17 mm rod more quickly than WT mice and exhibited a significant performance difference compared to shSCR control-treated animals 15 weeks after treatment. There was no significant difference in time to turn parameter (**E**) YAC128 mice treated with amiR136-A2 did not show any improvement in performance in either motor performance or learning capabilities on the rotarod test; mice treated with the low dose of amiRNA showed an improvement in time turn on the 17-mm rod 15 weeks after injection. P values are indicated by asterisks (* < 0.05; ** < 0.01; **** < 0.0001). For body weight 2-way ANOVA was employed and for the spleen weight 1-way ANOVA was used, both with the Tukey's test.

**Supplemental Table S1.** Oligonucleotides used for the generation of amiRNA constructs.

| Pri-miRNA shuttle | Insert | Oligonucleotide sequences 5'->3' |
|---|---|---|
| 451 | A2 | CCCAAGAAGCTCTCTGCTCAGCCTGTCACAACCTACTGACTGCCAGGGCACTTGGGAATGGCAAGGGCTGCTGCAGCTGCTGCTGCTGCAGCAGCT<br>GCAGCAGATCTTGCTATACCCAAGAAACGTGCCAGGAAGAGAACTCAGGACCCTGAAGCAGACTACTGGAAGGG |
| | G4 | CCCAAGAAGCTCTCTGCTCAGCCTGTCACAACCTACTGACTGCCAGGGCACTTGGGAATGGCAAGGGCTGCTGCGGCTGCGGCTGCTGCCGCAGC<br>CGCAGCAGATCTTGCCATTCCCAAGAAACGTGCCAGGAAGAGAACTCAGGACCCTGAAGCAGACTACTGGAAGGG |
| 136 | A2 | CACTCCACTGCCCGACGTCGCCTCGGTGGTGTTGGATGAGCCCTCGGAGGGCTGCTGCAGCTGCTGCTGCTCGATTCTTATGCTCGAGCAGCAGCA<br>GCTGCAGCAGTTCAGAGGGTTCTATCATTTCGTCGGATGGAAAGGAGTGTATTCTGAAGAT |
| | G4 | CACTCCACTGCCCGACGTCGCCTCGGTGGTGTTGGATGAGCCCTCGGAGGGCTGCTGCGGCTGCGGCTGCTCGATTCTTATGCTCGAGCAGCcGCA<br>GCcGCAGCAGTTCAGAGGGTTCTATCATTTCGTCGGATGGAAAGGAGTGTATTCTGAAGAT |
| 122 | A2 | GACAATGGTGGAATGTGGAGGTGAAGTTAACACCTTCGTGGCTACACCTTAGCAGAGCTGGCTGCTGCAGCTGCTGCTGCTTGTCTAAACTATAGC<br>AGCAGCAGCTGCAGCAGCCAGCTACTGCTAGGCTGTCTTGGCATCGTTTGCTTTGAGCAAGAAGGTTCATCT |
| | G4 | GACAATGGTGGAATGTGGAGGTGAAGTTAACACCTTCGTGGCTACACCTTAGCAGAGCTGGCTGCTGCGGCTGCGGCTGCTTGTCTAAACTATAG<br>CAGCCGCAGCCGCAGCAGCCAGCTACTGCTAGGCTGTCTTGGCATCGTTTGCTTTGAGCAAGAAGGTTCATCT |
| 155 | A2 | GCCTGGAGGCTTGCTTTGGGCTGTATGCTGGCTGCTGCAGCTGCTGCTGCTGTTTTGGCCACTGACTGACAGCAGCAGCAGCTGCAGCAGCCAGG<br>ACACAAGGCCCTTTATCAGCACTCACATGGAACAAATGGCCC |
| | G4 | GCCTGGAGGCTTGCTTTGGGCTGTATGCTGGCTGCTGCGGCTGCGGCTGCTGTTTTGGCCACTGACTGACAGCAGCcGCAGCcGCAGCAGTCAGG<br>ACACAAGGCCCTTTATCAGCACTCACATGGAACAAATGGCCC |

**Supplemental Table S2.** Off-targets with full complementarity to the A2 insert.

| Gene ID | Gene name | Localization | Expression in brain | Expression in other tissues |
|---|---|---|---|---|
| Golga4 | Golgi autoantigen, golgin subfamily a, 4 | ORF, 3' UTR* | Low** | High |
| Soga3 | SOGA family member 3 | ORF | High | Low |
| Maml1 | Mastermind like transcriptional coactivator 1 | ORF | Low | Low |
| Ccdc177 | Coiled-coil domain containing 177 | ORF | High | Low |
| Th | Tyrosine hydroxylase | ORF | High | Low |
| Ppp1r3f | Protein phosphatase 1, regulatory subunit 3F | ORF | Low | Low |

* 3'UTR in transcript ENSMUST00000212593.1. ** based on publication Guo S. et al., DOI: 10.1016/j.bbrc.2020.05.170

**Supplemental Table S3.** Sequences of primers used for RT-qPCR and genotyping.

| Gene | Primer orientation | Sequence |
|---|---|---|
| *β-actin* | F | AGAGCTACGAGCTGCCTGAC |
| | R | AGCACTGTGTTGGCGTACAG |
| *Cccdc177* | F | TCGGACAGGTAGAAAGAGCCAC |
| | R | CTGTTCCTGGCGGAAGCTCGA |
| *Cnr1* | F | ATCGGAGTCACCAGTGTGCTGT |
| | R | CCTTGCCATCTTCTGAGGTGTG |
| *Darpp32* | F | TCTCAGAGCACTCCTCACCAGA |
| | R | CACTCAAGTTGCTAATGGTCTGC |
| *Drd2* | F | CCTGTCCTTCACCATCTCTTGC |
| | R | TAGACCAGCAGGGTGACGATGA |
| *Gfap* | F | CACCTACAGGAAATTGCTGGAGG |
| | R | CCACGATGTTCCTCTTGAGGT |
| *Golga4* | F | GCAAATGGACCAGCAAGCAA |
| | R | GGGTTTTAGCGGAAGTCCCA |
| *HTT* | F | GTGCAGTGATGACGCAGAGT |
| | R | TCTTCGGGTCTCTTGCTTGT |
| *Htt* (for genotyping) | F | CCGCTCAGGTTCTGCTTTTA |
| | R | TGGACAGGGAACAGTGTTGG |
| *HTT* (for genotyping) | F | CCGCTCAGGTTCTGCTTTTA |
| | R | GGCTGAGGAAGCTGAGGAG |
| *Iba1* | F | TCTGCCGTCCAAACTTGAAGCC |
| | R | CTCTTCAGCTCTAGGTGGGTCT |
| *Maml1* | F | TCACAAGCAAGATGATGAGCACAG |
| | R | GCACGGAAGTCACTCCAGCA |
| *Ppp1r3f* | F | CCTGATGTTCGAGAGTCACTAGG |
| | R | TGCTGGTCAACATAACTTCGGGC |
| *Soga3* | F | AGATGGAGAAGCTGAGGGAAGAG |
| | R | AGTTGACAGGCATCCTCCTCGA |
| *Th* | F | GCCAAGGACAAGCTCAGGAA |
| | R | CTCAGTGCTTGGGTCAGGGT |

**Supplemental Table S4.** Antibodies used for Western blot analysis and immunohistochemistry.

| Protein | Dilution | Supplier | Secondary antibody |
|---|---|---|---|
| HTT (total) | 1:2000 in milk 5% PBS-T | Abcam (ab109115) | R-POX Jackson, ImmunoResearch 1:1000, milk 5% PBS-T |
| polyQ | 1:1000 in milk 5% PBS-T | Sigma-Aldrich (P1874) | M-POX Jackson, ImmunoResearch 1:1000, milk 5% PBS-T |
| plectin | 1:1000 in milk 5% PBS-T | Cell Signaling (#12254) | R-POX Jackson, ImmunoResearch 1:1000, milk 5% TBS-T |
| calnexin | 1:2000 in milk 5% PBS-T | Sigma-Aldrich (C4731) | R-POX Jackson, ImmunoResearch 1:1000, milk 5% PBS-T |
| HCN1 | 1:500 in milk 5% PBS-T | Abcam (ab176304) | R-POX Jackson, ImmunoResearch 1:1000, milk 5% PBS-T |
| RBM33 | 1:1000 in milk 5% PBS-T | Bethyl Laboratories (A303-926A) | R-POX Jackson, ImmunoResearch 1:1000, milk 5% PBS-T |
| HTT (aggregates) | 1:50 in 4% NGS PBS-T | Sigma-Aldrich (**MAB5374**) | Anti-mouse, Vector Laboratories (MP-7802) |
| NeuN | 1:500 in 4% NGS PBS-T | Millipore (MAB377) | Anti-mouse, Thermo Scientific, 1:1000, 4% NGS TBS-T |
| IBA1 | 1:1000 in 4% NGS PBS-T | WAKO (019-19741) | Anti-rabbit, Thermo Scientific, 1:1000, 4% NGS TBS-T |
| GFAP | 1:400 in 4% NGS PBS-T | Millipore (MAB3402) | Anti-mouse, Thermo Scientific, 1:1000, 4% NGS TBS-T |
| DARPP-32 | 1:500 in 4% NGS PBS-T | R&D Systems (MAB4230) | Anti-rat, Thermo Scientific, 1:1000, 4% NGS TBS-T |
